# System reconstruction of *Bacillus licheniformis* for efficient expression of alkaline protease

**DOI:** 10.1016/j.synbio.2026.07.005

**Published:** 2026-08-01

**Authors:** Qing Zhang, Mengyuan Zhang, Zhihao Zhu, Haijing Ma, Fei Mo, Jiaqi Lei, Shisi He, Yongqing Liao, Shihao Yan, Dongbo Cai, Shouwen Chen

**Affiliations:** a State Key Laboratory of Biocatalysis and Enzyme Engineering, Hubei Engineering Research Center for Microbial Cell Factories, College of Life Sciences, Hubei University, Wuhan, 430062, China; b Hubei Jiangxia Laboratory, Wuhan, 430200, China

**Keywords:** *Bacillus licheniformis*, Alkaline protease, Codon optimization, Genome reduction, Modular engineering, Energy metabolism

## Abstract

Industrial enzymes are widely used in diverse applications, but low productivity limits their further widespread utilization. This research aimed to develop high-performance alkaline protease (AprE) expression strains of *Bacillus licheniformis* through element optimization and modular engineering. Firstly, the *aprE* gene expression cassette was systematically optimized through element engineering. To minimize host background interference, five large gene fragments were deleted from the genome of *B. licheniformis* DW2. This expression cassette and genome-reduced strain resulted in 5.77-, 4.84- and 1.31-fold increases in the activities of alkaline protease, nattokinase and chitinase, respectively. Crucially, metabolomics analysis then served as the pivotal discovery tool, revealing that high expression of AprE was constrained by insufficient precursor amino acids and excessive metabolic overflow. Subsequently, the amino acid biosynthesis, energy metabolism, overflow metabolism, and cell membrane/wall modules of the strain were successively modified. The final AprE expression host DM6E10 achieved a remarkable enzyme activity of 34,343 U/mL, with a maximum activity of 107,100 U/mL in a 5-L bioreactor. This study built an efficient cell factory for AprE production and provided insights for the optimization of other protein expression hosts.

## Abbreviations

AprE: Alkaline protease
UTR: Untranslated regions
GRAVY: Grand average of hydropathicity
SDS-PAGE: Sodium dodecyl sulfate - polyacrylamide gel electrophoresis
GFP: Green fluorescent protein
ATP: Adenosine triphosphate
NK: Nattokinase
GABA: γ-aminobutyric acid
GC-MS: Gas chromatography mass spectrometry
TCA cycle: Tricarboxylic acid cycle
cyt c: cytochrome c
PCA: Principal component analysis
Acyl-CoA: Acyl coenzyme A
FAS II: Type II fatty acid synthase system
Acyl-ACP: Acyl-acyl carrier protein
Acyl-P: Acyl phosphate
LPA: Lysophosphatidic acid
PA: Phosphatidic acid
CDP-DAG: Cytidine diphosphate diacylglycerol
PGP: Phosphatidylglycerol phosphate
PG: Peptidoglycan
CL: Cardiolipin
CP: Carboxypeptidase
LTA: Lipoteichoic acid

## 1 Introduction

Industrial enzymes are extensively utilized in diverse sectors, including the food industry, detergent formulations, and animal feed [1]. According to Grand View Research (2023), the global industrial enzyme market was valued at $7.53 billion in 2023 and is projected to reach $12.64 billion by 2033 [2]. Alkaline protease (AprE) boasts a multibillion-dollar market and is widely applied in biological detergents for low-temperature decontamination, eco-friendly leather processing, and food proteolytic hydrolysis. Besides, with the increasing global commitments to carbon neutrality, the replacement of conventional chemical manufacturing with enzymatic biocatalysis has emerged as an essential technological transition.

Efficient enzyme expression hinges on two critical elements, the enzyme and its expression host [3]. For enzyme engineering, strategies such as directed evolution, rational design and AI-assisted protein evolution have been shown to effectively enhance enzyme properties and produce commercially valuable proteins [[4], [5], [6]]. For instance, the G95P substitution obtained by error-prone PCR significantly enhanced both catalytic activity and stability of AprE from *Bacillus clausii* [5]. In host engineering, removing genes for competing products, nonessential proteins, or poorly characterized proteins can optimize the host genome and thus enhance recombinant protein expression [7].

Synthetic biology and metabolic engineering provide powerful approaches for constructing high-performance expression hosts. The optimization of codons [8] and expression elements, including promoters, UTRs, and pro-peptides [9], is crucial for constructing efficient gene expression cassettes. In addition, genome streamlining [10], deletion of competitive pathways [11], enhancement of precursor supply [12], and cell membrane and cell wall engineering [13] are beneficial for optimizing protein expression strains. While existing research has extensively explored individual modules affecting protein biosynthesis, it is crucial to further investigate the balance between the supply and consumption of precursors. Additionally, identifying the key limiting factors will provide guidance for targeted modifications of metabolic pathways.

*Bacillus species*, particularly *Bacillus subtilis*, *Bacillus licheniformis* and *Bacillus amyloliquefaciens*, are primary hosts for industrial enzyme production. Recently, efficient expression of various target proteins (e.g. α-amylases, protease, pullulanase) in *B. subtilis* has been achieved through engineering of expression elements and hosts [14,15]. Previously, modular engineering strategies implemented in *B. amyloliquefaciens* BAΔupp have been reported to enhance AprE production by 39.6% [16]. As a powerful and indispensable non-model microorganism for applied and industrial microbiology, *B. licheniformis* possesses advantageous features, including good secretory capacity, strong robustness, and simple genetic manipulation, making it a competitive host for industrial enzyme expression (e.g. alkaline proteases, nattokinase) [17,18]. However, the biological breeding and industrial application of *B. licheniformis* still face challenges, such as the unclear metabolic mechanisms and the imbalanced distribution of metabolic fluxes.

In this study, we aimed to systematically optimize *B. licheniformis* DW2 for protein expression, employing AprE from *B. clausii* as the reporter protein. The *aprE* gene expression cassette was first designed and optimized through element engineering. Subsequently, the DW2 genome was reducted to minimize the host background interference and obtain a universal expression host. Thereafter, the limiting factors of AprE high expression were identified based on metabolomic analysis. Finally, *B. licheniformis* DW2 was reconstructed through enhancing the supply of amino acids and energy precursors, modulating cell membrane and cell wall, and reducing overflow metabolism. Overall, this research not only established an efficient protein expression platform in *B. licheniformis* but also provided broadly applicable strategies for the development of other industrial enzyme chassis.

## 2 Material and methods

### 2.1 Strains and cultivation conditions

All strains used in this study were listed in Table S1. The plasmids and primers used in this study were presented in Table S2 and Table S3, respectively. *E. coli* DH5α was used for plasmid construction, and *B. licheniformis* DW2cas9n was used as the parental strain. Gene expression, knock-out, and integration vectors were constructed basing on the shuttle vector pHY300PLK and T_2_ (2)-Ori, respectively.

*B. licheniformis* and *E. coli* were grown on Luria-Bertani (LB) medium or in LB broth supplemented with antibiotics (tetracycline 20 μg/mL; kanamycin 20 μg/mL). AprE fermentation medium contained 50 g/L corn starch, 45 g/L soybean meal, 6 g/L CaCO_3_, 5 g/L (NH_4_)_2_SO_4_, and 0.35 g/L thermostable amylase. TB medium contained 12 g/L peptone, 24 g/L yeast extract, 5 g/L glycerol, 16.42 g/L K_2_HPO_4_, 2.31 g/L KH_2_PO_4_. The nattokinase fermentation medium consisted of 20 g/L glucose, 10 g/L peptone, 5 g/L yeast extract, 10 g/L NaCl, 10 g/L soy peptone, 10 g/L corn steep liquor, and 6 g/L (NH_4_)_2_SO_4_. The chitinase fermentation medium contained 50 g/L corn starch, 45 g/L soybean meal, 6 g/L CaCO_3_, and 5 g/L (NH_4_)_2_SO_4_. All cultures were maintained at 37°C unless otherwise stated.

### 2.2 Construction of recombinant strains

The construction of gene expression strains was referred to our previously reported method [19]. Plasmid pHY300PLK was used to construct a series of AprE expression vectors. The construction procedure of AprE expression vector pHY-P43-aprE served as an example, promoter P43 from *B. licheniformis* DW2, signal peptide of alkaline protease SP_AprE_ and gene *aprE* from *B. clausii* were amplified by corresponding primers (Table S3), and then fused by splicing overlap extension (SOE)-PCR, and inserted into pHY300PLK using ClonExpress One Step Cloning Kit (Vazyme Biotech Co., Ltd, Nanjing, China), resulting in the plasmid pHY-P43-aprE. Besides, the double- and multi-mutant plasmids were constructed based on the plasmid pHY-P43-aprE. Then, these plasmids were transferred into *B. licheniformis* DM1 to obtain corresponding recombinant strains. Similarly, other protease and GFP expression strains were obtained with the same method.

Temperature-sensitive plasmid T_2_ (2)-Ori was used for constructing the gene integration and promoter replacement vectors. Taking vector T_2_-△oxdC as an example, the upstream and downstream homology arm of gene *oxdC* from *B. licheniformis* were amplified respectively, and after Splicing Overlap Extension (SOE)-PCR, they were assembled into T_2_ (2)-Ori vector, generating T_2_-△oxdC (Table S2). The generations of gene deletion, integration and promoter replacement strains were obtained as previous described [20].

CRISPR/Cas9n was conducted for the deletion of large segments from DW2 genome, and CRISPR/dCas9 was used for gene inference, and the relative methods were referred to the previous literature [21]. The gRNA expression plasmids were electroporated into *B. licheniformis* cells, and the deleted genes were verified by DNA sequencing.

### 2.3 Alkaline protease fermentation and enzyme activity detection

The recombinant strain of *B. licheniformis* was cultivated on LB agar plate for 12–14 h, and a single colony was first transferred to 5 mL LB medium, then scaled up to 20 mL to obtain the seed solution (OD_600_ ≈ 4.0). The seed culture was inoculated into 250 mL Erlenmeyer flask containing 20 mL AprE fermentation medium at a 5% inoculum dosage, and cultivated for 48 h to reach the fermentation end point (characterized by pH 8.3). Subsequently, the supernatant was obtained after centrifugation at 12,000 rpm for 10 min at 4°C, and then diluted serially. The AprE activity was detected using the Folin-Ciocalteu reagent according to Chinese National Standard GB/T 23,257-2009. One unit (U) of AprE activity was defined as the amount of enzyme producing 1 μg tyrosine per minute under specified conditions (40°C, pH10.5) through casein hydrolysis. The molecular mass of denatured recombinant protein was measured by sodium dodecyl sulfate-polyacrylamide gel electrophoresis (SDS-PAGE) with protein markers (Vazyme, China).

### 2.4 Methods for metabolomic analysis

Each AprE fermentation flask was rapidly cooled to 9 ± 2°C using liquid nitrogen to quench the cells. Cold 0.85% NaCl (v/v) was added to dilute the fermentation broth twice. After centrifugation at 1500 rpm at 4°C for 15 min, the supernatant was obtained. After centrifugation, the cells were washed with cold 0.85% NaCl and 2.5% (NH_4_)_2_CO_3_ using high-speed centrifugation (10,000 rpm) at 4°C for 5 min. The extraction procedure was carried out according to Yuan et al. [22]. Afterward, five OD_600_ units of cells were used for metabolites extraction and GC-MS analysis. GC-MS data were first converted into *abf* format using Reifycs Abf Converter (Analysis Base File), and the transformed data was matched with Fiehn Bin Base Metabolome Database using MSDIAL software, generating an output file designated as “Height file”. The peak area of each metabolite was normalized with internal standard (glycolipid concentrate mixture, Fatty acid methyl esters, C8–C30). The contents of intracellular organic carboxylic acid were analyzed according to the results of GC-MS. Subsequently, the processed data was subjected to principal component analysis (PCA). And SIMCA 14.1 (Umetrics, Sweden) was used to perform orthogonal partial least squares discriminant analysis (OPLS-DA). Metabolites with a VIP value > 1, *p* value < 0.05, and a fold change of >2 or <0.5 in each comparison were selected as differential metabolites. Analysis of differential metabolite's contributions to the Kyoto Encyclopedia of Genes and Genomes (KEGG) pathway was performed using MetaboAnalyst software (http://www.metaboanalyst.ca/MetaboAnalyst/). Finally, a heat map was generated using HemI (Heatmap Illustrator, http://hemi.biocuckoo.org/).

### 2.5 Methods of restriction codons analysis

First, the ideal proportion of codons (Ideal Prop., ‰; Table S6) was derived from the synonymous codon usage of *B. licheniformis*. We calculated the actual proportion of each codon (Codon Usage, ‰) within the exogenous *aprE* gene. Restriction codons were defined as those codons meeting both criteria based on our predetermined threshold criteria: (1) ideal proportion below 5‰ (in permillage, ‰), and (2) actual proportion exceedingly twice this ideal proportion.

### 2.6 Quantitative real-time PCR

The methods of RNA extraction and transcriptional level analysis were optimized based on previously described protocols [23]. Cells in middle log-phage were harvested for mRNA isolation, and RNA was extracted using the Bacterium Total RNA Extraction Kit (APEXBIO, China). The cDNA was produced using the HiScript®II Q RT SuperMix kit (Vazyme, China). Gene expression analysis of the target genes was performed using a CFX96 Real-Time PCR System (Bio-Rad, Hercules, CA, USA), and all assays were performed with three biological replicates with technical triplicates. The 16S rRNA gene was the reference gene, and the fold changes were calculated using 2^−ΔΔCT^ method [24].

### 2.7 Analytical methods

Biomass was evaluated by measuring the absorbance at 600 nm (OD_600_) using a spectrophotometer (UV-160A; Shimadzu, Japan). Residual glucose was detected using biosensor analyzer (SBA 40C, Shandong Academy of Sciences, China) with an immobilized enzyme membrane. For measurements of free amino acid concentrations, samples were first centrifuged at 4000 rpm, 4°C for 5 min to remove solid precipitates from the medium, then centrifuge the supernatant at 12,000 rpm, 4°C for 10 min to obtain bacterial precipitates. The supernatant was derivatized according to the method described by Zhan et al. and subsequently analyzed by gas chromatography [25]. The concentrations of acetoin, 2, 3-butanediol and acetate were determined by gas chromatography according to Wang et al. [26]. The intracellular ATP concentration was determined by ATP assay Kit (Beyotime Biotechnology, China), according to its instruction manual. Established methods were applied for cell permeability and anion exchange capacity, as reported by Mo et al. [13]. The net charge and isoelectric point of signal peptide were calculated using an online tools – peptide property calculator (https://www.novopro.cn/tools/calc_peptide_property.html). Nattokinase activity was measured by fibrin degradation [27]. The chitinase activity was determined by DNS method using colloidal chitin as the substrate [28].

### 2.8 AprE production in 5 L bioreactor

For AprE production in a 5 L bioreactor, *B. licheniformis* strain was cultivated in 2.5 L AprE production medium. The AprE production medium contained 30 g/L glucose, 40 g/L corn pulp, 15 g/L yeast extract, 5 g/L (NH_4_)_2_SO_4_, 1 g/L MgSO_4_, 6 g/L CaCO_3_ and 1 g/L K_2_HPO_4_. The fermentation parameters were as follows: the inoculum volume was 10%, with antibiotics added at 3‰. The pH was controlled at 7.0 throughout the fermentation. The initial aeration rate was 2 L/min, increasing to a maximum of 7 L/min. The initial agitation rate was 200 rpm, with a maximum of 800 rpm. The dissolved oxygen was kept at 25% ± 5% by adjusting agitation rate and aeration.

For fed-batch fermentation, the initial glucose concentration was reduced to 30 g/L, 10 g/L Glucose was added when the glucose concentration fell below 10 g/L and the cell wet weight was less than 90. When the cell wet weight exceeded 90, a total of 200 g corn starch was added. The feed of nitrogen source was to fill 50 g corn pulp with distilled water to 150 mL, after sterilization, and to make a one-time supplement at the 12 h and 24 h of fermentation respectively.

### 2.9 Statistical analysis

All samples were analyzed in triplicate, and the data were presented as the mean ± the standard deviation for each sample point. Data analysis was done using one-way analysis of variance (ANOVA) and Duncan's multiple range using SPSS® 19.0 at *P* ≤ 0.05.

## 3 Results

### 3.1 Optimization of expression cassettes via element engineering

#### 3.1.1 Codon optimization of aprE based on comparative analysis

**Fig. 1:**
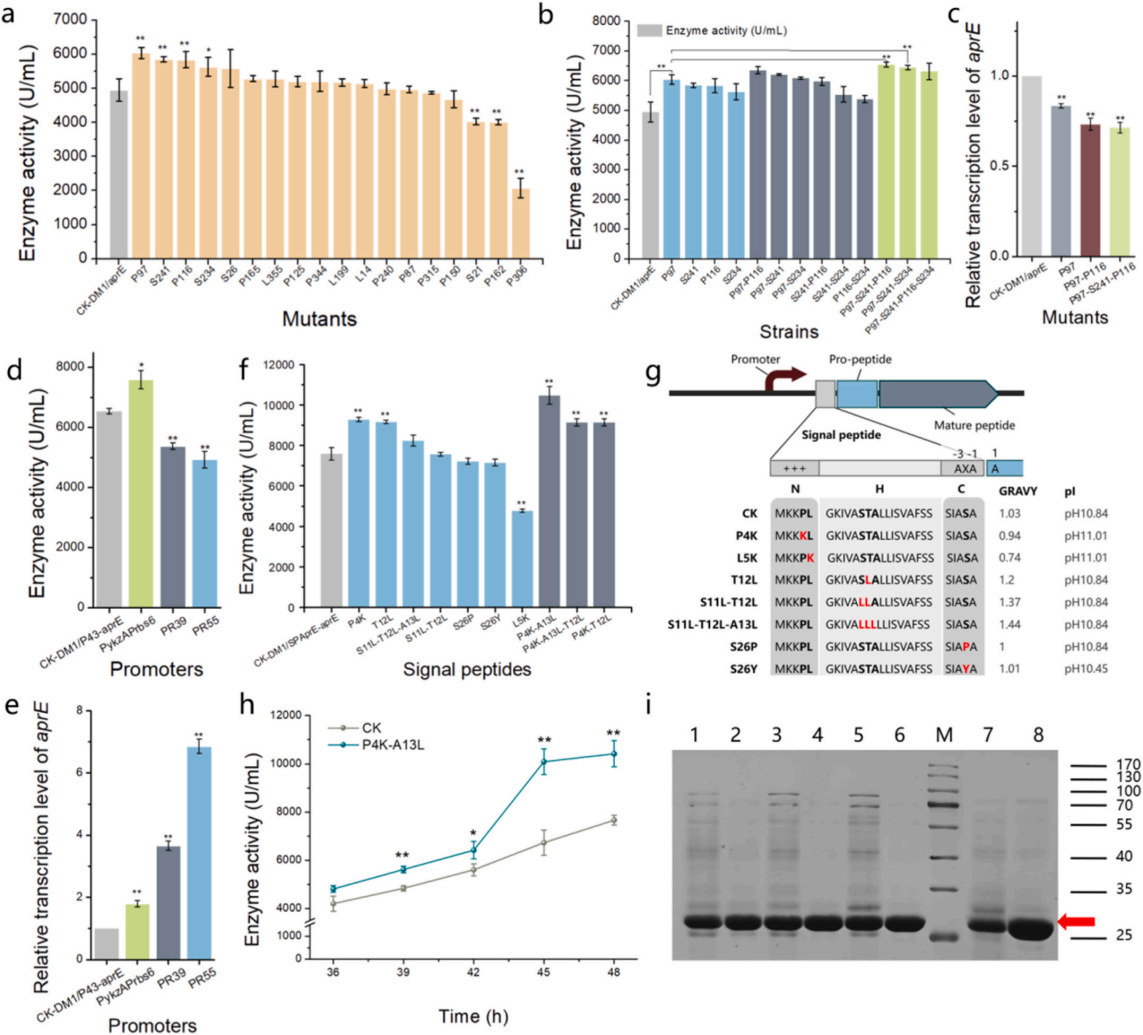
Effects of element optimization on AprE expression. **a:** Activities of codon signal-point mutants. **b:** Activities of codon multi-point mutants. **c:** The relative transcriptional level of *aprE* in codon mutant strains. **d:** AprE activities of promoter mutants. **e:** The relative transcriptional level of *aprE* mediated by different promoters. **f:** AprE activities of signal peptide mutants. **g:** Isoelectric point (pI) and GRAVY values of signal peptide mutants. **h:** AprE activity change curves of the mutants P4K-A13L and CK. **i:** SDS-PAGE analysis of mutants P4K-A13L and CK. The red arrow indicates AprE, lane 1, 36 h-CK; Lane 2, 36 h P4K-A13L; Lane 3, 39 h-CK; Lane 4, 39 h P4K-A13L; Lane 5, 42 h-CK; Lane 6, 42h P4K-A13L; Lane 7, 45h-CK; Lane 8, 45h P4K-A13L; M, protein Marker. Data are represented as the means of three replicates, and error bars represent the standard deviations. *, *P* < 0.05; **, *P* < 0.01; ***, *P* < 0.001 indicate the significance levels between control and its mutant strains.

To optimize the codons of *aprE* gene, we calculated the actual proportion of each codon, accounting for differences in codon usage bias between the phylogenetically close microorganisms *B. licheniformis* and *B. clausii* (Tables S4–S6). This study identified codons potentially limiting *aprE* expression in *B. licheniformis*, including proline codon CCA, leucine codon CUA, and serine codon AGU. Subsequently, these codons were replaced with preferred synonymous codons, and the resulting 18 mutants were constructed and expressed in strain DM1 (DW2*cas9n*△M44) (Fig. 1a). The results of activity assays revealed that mutants P97^CCG^, P116^CCG^, S234^TCG^ and S241^TCG^ exhibited increased activity compared to control strain DM1/aprE (CK), with enhancements of 22.02%, 18.08%, 17.87% and 13.71%, respectively (Fig. 1a). Conversely, the activities of mutants S21^TCG^, P162^CCG^ and P306^CCG^ were significantly decreased by 18.59%, 18.91% and 58.14%, respectively. Enzyme activity changes at the remaining sites were not statistically significant.

Subsequently, the favorable mutation sites P97^CCG^, P116^CCG^, S234^TCG^ and S241^TCG^ were combined. Among the six double-mutant constructs generated, P97^CCG^-P116^CCG^ exhibited the highest activity, representing a 28.40% increase over the control strain DM1/aprE (CK) (Fig. 1b). The triple-mutant P97^CCG^-S241^TCG^-P116^CCG^ also demonstrated enhanced activity, achieving a 32.11% increase compared to CK. Furthermore, RT-qPCR analysis revealed that transcriptional level of gene *aprE* progressively decreased with the serial combination of optimized codons, culminating in a significant 28.50% reduction in triple-mutant strain compared to control (Fig. 1c). We indicated that the optimized codons may have influenced the transcriptional level of genes by introducing secondary structures or triggering other physiological activities. However, the substitution of restrictive codons may also have reduced the retention of ribosomes on mRNA, enhancing translation efficiency, and the specific mechanism still requires further investigation.

#### 3.1.2 Promoter engineering of the aprE gene

To further optimize the expression cassette, we evaluated four promoters (P43, P_ykzA_P_rbs6_, PR55 and PR39) previously constructed in our laboratory for *aprE* expression [29]. The results of AprE activity demonstrated that P_ykzA_P_rbs6_ exhibited the best performance, AprE activity driven by which was increased by 16.06% compared to P43, achieving 7595 ± 309 U/mL (Fig. 1d), and RT-qPCR analysis revealed that the transcriptional level of *aprE* mediated by P_ykzA_P_rbs6_ was 79.51% higher than that of P43 (Fig. 1e). Interestingly, while stronger promoters like PR55 further increased transcription levels, they resulted in moderate decreases in recombinant protein expression, suggesting potential bottlenecks in translation, protein folding, or secretion processes.

#### 3.1.3 Enhancing secretion through optimizing the tripartite structure of signal peptide

To improve the secretion efficiency of AprE, we analyzed the tripartite structural characteristics (N-, H-, C- regions) of native Sec-type signal peptide SP_AprE_. Based on the expression cassette P_ykzA_P_rbs6_-SP_AprE_-aprE, seven SP_AprE_ mutants were constructed, and AprE activity of mutant P4K was increased by 22.16%, compared with the original signal peptide strains (DM1/P_ykzA_P_rbs6_-SP_AprE_-aprE), and dominant mutant P4K-A13L further enhanced performance, achieving an activity of 10,471 ± 444 U/mL, increased by 37.86% (Fig. 1f). Biophysical characterization showed that the optimized signal peptide P4K-A13L exhibited an increased net positive charge (from +3.0 to +4.0 at pH 7.0) and a higher GRAVY value (grand average of hydropathicity, from 1.03 to 1.19), which increased the hydrophobicity of signal peptide (Fig. 1g). Fermentation profiling confirmed that AprE activity and extracellular protein content of optimal mutant P4K-A13L were significantly higher than controls throughout the whole fermentation process (Fig. 1h–i). Therefore, through codon optimization and element engineering, we obtained a superior expression cassette P_ykzA_P_rbs6_-SP_AprE-P4K-A13L_-aprE^P97-S241-P116^.

### 3.2 Construction of enzyme-expression chassis of *B. licheniformis* DW2 through genome streamlining

#### 3.2.1 Effect of large fragment deletion on AprE expression

**Fig. 2:**
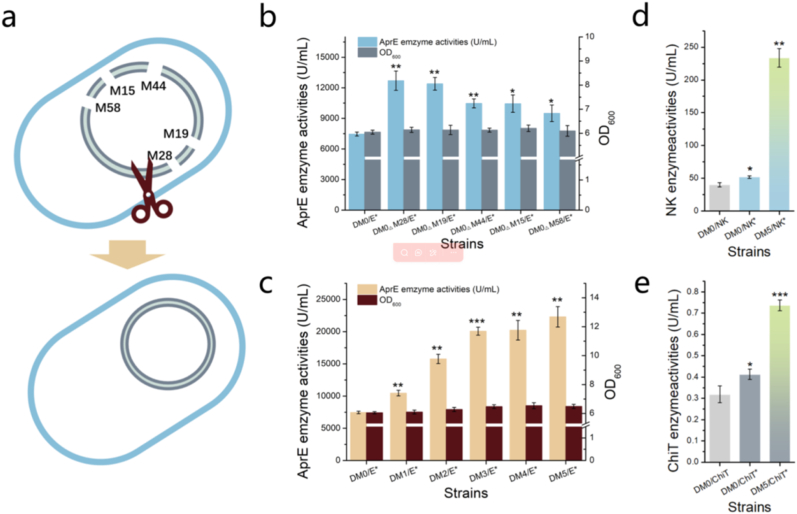
Effect of genome reduction on protein expression. **a:** Schematic illustration of genome reduction. **b:** AprE activities and biomass of large fragment deletion strains. **c:** AprE activities and biomass of large fragment superposition deletion strains. **d:** Nattokinase activities of strains DM0/NK, DM0/NK* and DM5/NK*. **e:** Chitinase activities of strains DM0/ChiT, DM0/ChiT* and DM5/ChiT*. Data are represented as the means of three replicates and error bars represent the standard deviations. *, *P* < 0.05; **, *P* < 0.01; ***, *P* < 0.001 indicate the significance levels between control and its mutant strains.

Excessive genomic redundancy can impair gene expression and product synthesis [30]. In this study, based on the genome sequencing results of DW2, we selected five large gene fragments, screened redundant genes, and studied their influence on AprE expression (Table S6). The deletion of fragments M44, M19, M28, M15, and M58 from DM0 (DW2*cas9n*) was conducive to AprE expression, and their deletion had no obvious effects on cell growth (Fig. 2a–b). Specifically, M44, M15, and M58 encode proteins involved in the synthesis of competitive metabolites—bacitracin, lichenysin, and poly γ-glutamic acid (γ-PGA), respectively. M19 corresponds to a prophage region gene island, while the M28 region, located at positions 350,347-370,441, comprises a complex set of genes (Table S7). Notably, the deletion of M28 led to the most substantial enhancement in AprE activity (Fig. 2b).

Superimposed deletion of fragments M44, M19, M28, M15, and M58 generated derivative strains DM1, DM2, DM3, DM4, and DM5, and then the optimized expression vector pHY-P_ykzA_P_rbs6_-SP_AprE-P4K-A13L_-aprE^P97-S241-P116^ (denoted as E*) was electroporated into these strains (Fig. 2c). Specifically, as multiple fragments were sequentially deleted, AprE activity gradually increased, and strain DM5/E* achieved an activity of 22,295 ± 1580 U/mL, representing a 1.99-fold increase over DM0/E* (Fig. 2c), and a 5.77-fold increase compared to DM0/E. Furthermore, DM5/E* exhibited higher biomass, after 24 h fermentation in TB medium, its biomass (6.49 ± 0.14) was 7.27% higher than that of DM0/E* (6.05 ± 0.08) (Fig. 2c).

Subsequently, the preferred expression element and the genome reducted host DM5 exhibited excellent performance in producing alikaline protease, nattokinase (NK) from *B. subtilis* and chitinase (ChiT) from *Caldicellulosiruptor acetigenus*. First, the original elements (promoters and signal peptides) were replaced with preferred expression element combination P_ykzA_P_rbs6_-SP_AprE-P4K-A13L_, and then the resulting plasmids pHY-P_ykzA_P_rbs6_-SP_aprE-P4K-A13L_-NK (abbreviated as NK*) and pHY-P_ykzA_P_rbs6_-SP_aprE-P4K-A13L_-ChiT (ChiT*) were electroporated into the DM0 strain, the enzyme activities of DM0/NK* and DM0/ChiT* were increased by 29.50% and 29.74%, respectively (Fig. 2d–e). Furthermore, the optimized vectors were transferred into genome-reducted host DM5, and the enzyme activities of DM5/NK* and DM5/ChiT* were increased by 351.09% and 78.37%, respectively, compared to the controls DM0/NK* and DM0/ChiT* (Fig. 2d–e). These results also indicated that the combined use of expression elements and the reducted host DM5 provides an effective platform for enhancing the secretory expression of diverse industrial enzymes.

### 3.3 Metabolomics-assisted analysis of the limiting factors on AprE production

#### 3.3.1 Metabolomics analysis of AprE strains with differential expression level

**Fig. 3:**
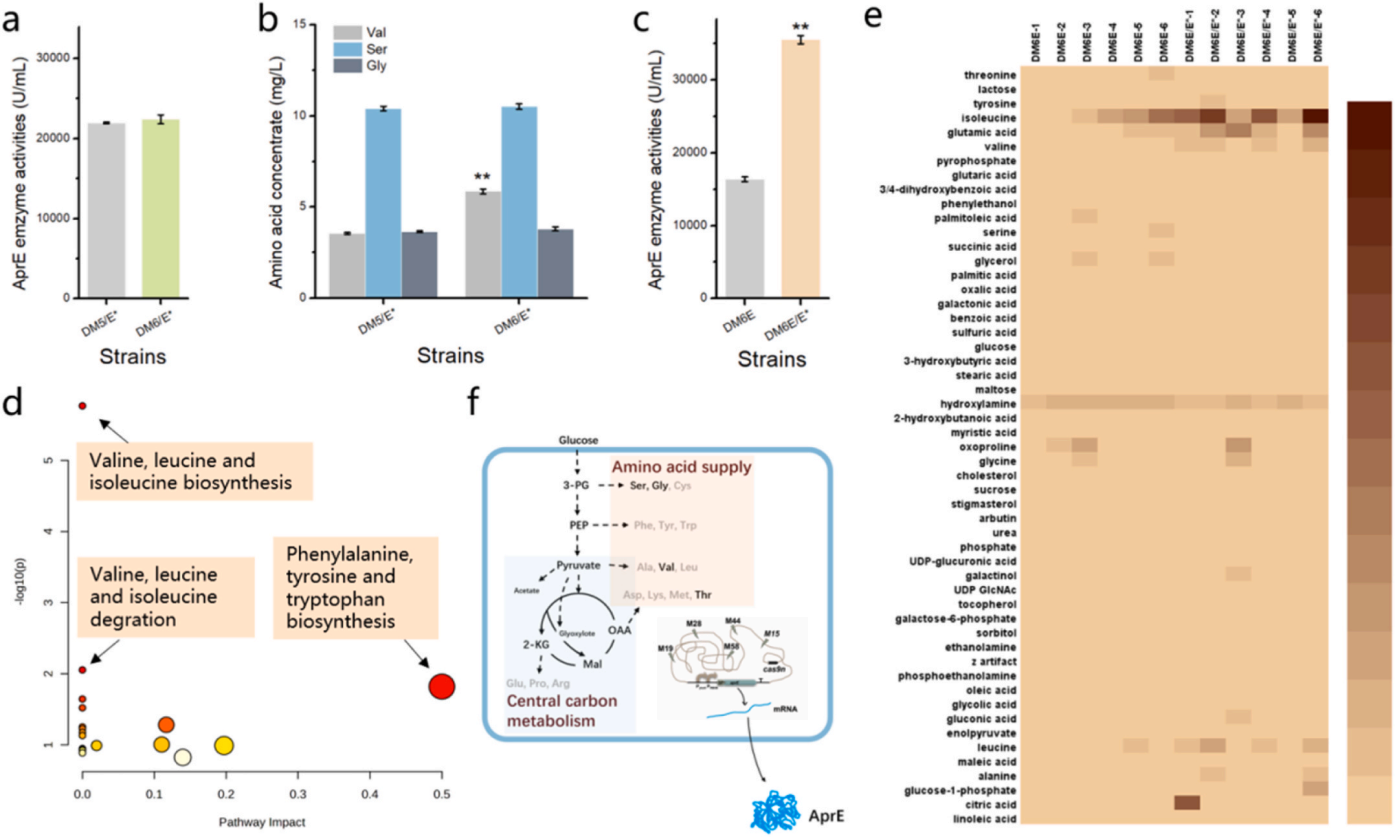
Metabolomics analysis of customized AprE expression chassis. **a:** AprE activities of DM5/E* and DM6/E*. **b:** The amino acid concentrations of DM5/E* and DM6/E*. **c:** AprE activities of DM6E and DM6E/E*. **d:** Differential metabolic pathways. **e:** Differential metabolites. **f:** Schematic illustration of the limiting factors. Data are represented as the means of three replicates and error bars represent the standard deviations. *, *P* < 0.05; **, *P* < 0.01; ***, *P* < 0.001 indicate the significance levels between control and its mutant strains.

The amino acid composition analysis revealed that branched-chain amino acids (BCAAs) constitute the main components of AprE. Gene *bcd,* encoding leucine dehydrogenase, is responsible for the degradation of BCAAs, so *bcd* was deleted from DM5 to redirect metabolic flux toward BCAA biosynthesis, attaining strain DM6. The AprE expression strain DM6/E* was constructed simultaneously. As shown in Fig. 3, AprE activity of DM6/E* was 22,408 ± 562 U/mL, which was 2.00% higher, and the valine concentration of DM6/E* was increased by 64.90% to 5.87 ± 0.22 mg/L, compared to DM5/E* (3.57 ± 0.14 mg/L) (Fig. 3b). The marginal improvement in enzyme activity despite substantial valine accumulation suggests the existence of rate-limiting bottlenecks beyond precursor availability.

Consequently, customized hosts with differential AprE expression levels were constructed for metabolomic analysis. The AprE expression cassette P_ykzA_P_rbs6_-SP_AprE_-aprE was integrated into the DM6 genome to generate DM6E. Strain DM6E/E* was obtained by transferring pHY-P_ykzA_P_rbs6_-SP_AprE-P4K-A13L_-aprE^P97-S241-P116^, into DM6E. Their AprE activities were 15,232 ± 325 U/mL and 35,494 ± 547 U/mL, respectively (Fig. 3c). Furthermore, a metabolomic analysis of DM6E and DM6E/E* was performed, which quantitatively profiled a total of 53 metabolites. Seven differential metabolites was identified through SIMCA analysis; notably, the threonine content in DM6E/E* was significantly decreased compared to the control, while the remaining six metabolites exhibited increases (Table 1). Additionally, the coordinated accumulation of amino acid precursors (valine, isoleucine, tyrosine, and glutamate) and the energy carrier (pyrophosphate) suggested adaptive metabolic remodeling that supports high-level AprE expression (Fig. 3e–Table 1). Specifically, the concentrations of overflow metabolites in central carbon metabolism, including glycolic acid, ethanolamine and oxalic acid, were relatively higher (Table S8). These results indicate that insufficient precursor amino acids and excessive metabolic overflow are critical limitations for achieving high levels of AprE production (Fig. 3f).

**Table 1:** Differential metabolites of strains DM6E and DM6E/E*.

Metabolites	KEGG	DM6E ave	DM6E/E* ave	*p-*value	(DM6E/E*)/DM6E
Threonine	C00188	4.966 ± 2.686	1.953 ± 1.368	4.23E-02	0.393
Lactose	C00243	2.278 ± 0.777	4.731 ± 1.379	5.42E-03	2.077
Tyrosine	C00082	2.280 ± 1.008	5.015 ± 2.461	4.16E-02	2.200
Isoleucine	C00407	21.959 ± 19.843	66.244 ± 35.168	2.80E-02	3.017
Glutamic acid	C00025	5.205 ± 5.502	26.740 ± 18.579	3.53E-02	5.137
Valine	C00183	1.426 ± 1.691	9.447 ± 6.317	2.53E-02	6.626
Pyrophosphate	C00013	0.357 ± 0.799	2.392 ± 1.850	4.35E-02	6.705

#### 3.3.2 Optimization of the supply of amino acid precursors for AprE synthesis in DM6E

**Fig. 4:**
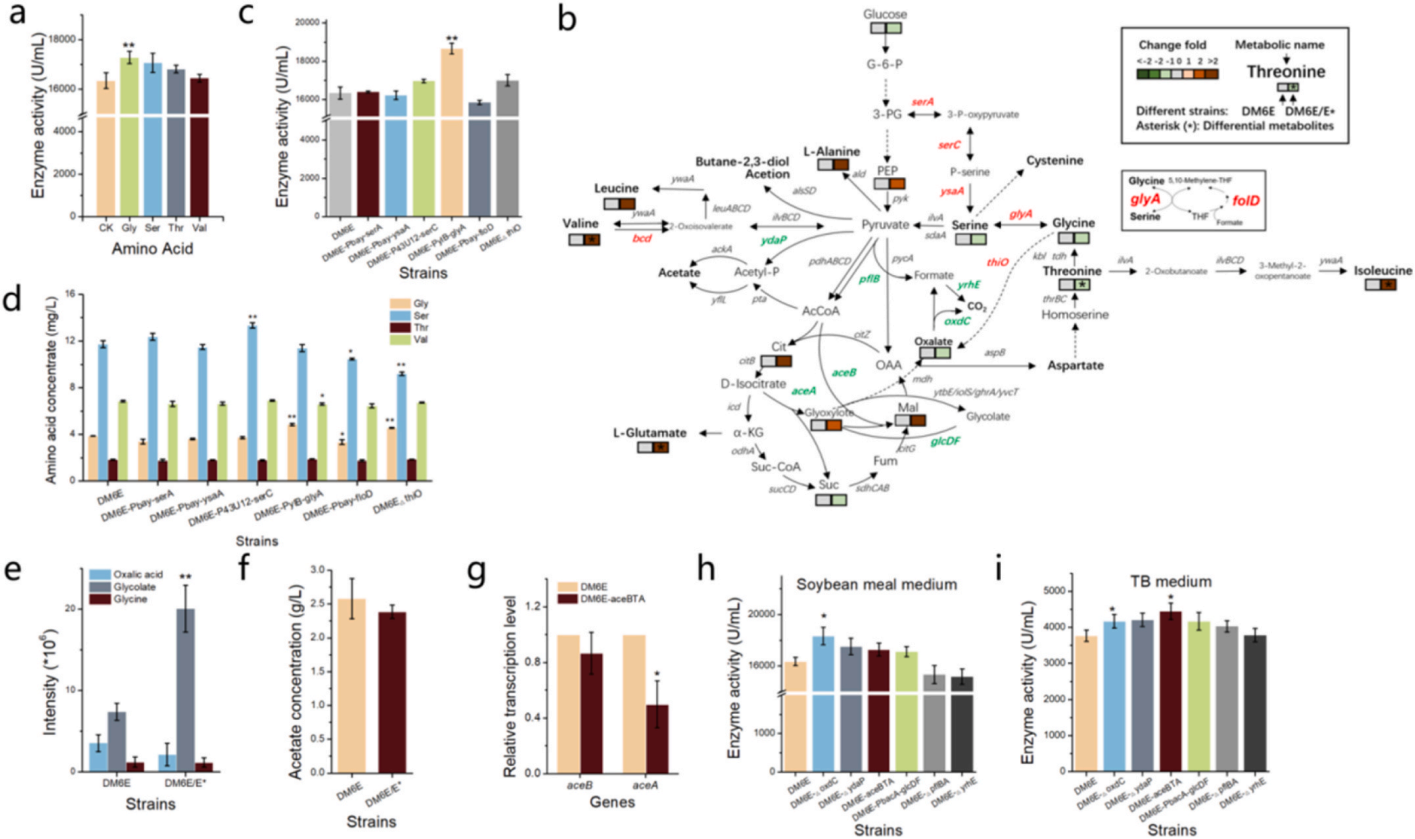
Optimization of precursor amino acids supply for AprE expression. **a:** Effect of amino acid addition on AprE activity of DM6E. **b:** Relative concentrations of intracellular metabolites in DM6E and DM6E/E*. The ratios of each metabolite in the strains DM6E/E* and DM6E are represented by varying colors, with the different metabolites marked with *. Genes in the amino acid biosynthesis module are marked in red, and those in the central carbon metabolism module are highlighted in green. **c:** Effect of amino acid synthesis pathway modification on AprE activity. **d:** Intracellular concentrations of amino acid. **e:** The relative contents of oxalic acid, glycolate and glycine of DM6E and DM6E/E*. **f:** Acetate concentrations. **g:** Relative transcription levels of *aceB* and *aceA* in DM6E7 and DM6E7-aceBTA. **h:** AprE activities of reduced overflow metabolism engineering strains in AprE fermentation medium. **i:** AprE activities in TB medium. Data are represented as the means of three replicates and error bars represent the standard deviations. *, *P* < 0.05; **, *P* < 0.01; ***, *P* < 0.001 indicate the significance levels between control and its mutant strains.

To address limitations in precursor amino acid availability, glycine, serine, threonine, and valine with final concentrations of 0.03 g/L were added separately into medium at 24 h. Fermentation results revealed that glycine supplementation significantly enhanced AprE activity, whereas supplementation with other amino acids had negligible effects (Fig. 4a). Integrating these findings with the biosynthetic pathways of the respective amino acids (Fig. 4b), we sequentially engineered the strain by overexpressing *serA*, *ysaA*, *serC*, *glyA*, and *folD*, and by deleting *thiO*. The overexpression was achieved by promoter engineering, wherein the native promoters of *serA*, *ysaA*, and *folD* were replaced with Pbay, while those of *serC* and *glyA* were substituted with P43U12 and PylB, respectively.

Fermentation analysis showed that AprE activity of strain DM6*E*-PylB-glyA (DM6E1) reached 18,665 ± 276 U/mL, which was a 14.17% increase over the control strain DM6E (Fig. 4c). Meanwhile, the intracellular glycine content of DM6E1 achieved a 25.75% elevation (Fig. 4d), suggesting that *glyA* overexpression enhances glycine availability and thereby boosts AprE activity. This enhancement may be attributed to improved cell membrane/wall permeability, and increased thermal stability of the target protein [31]. While other genetic modifications similarly elevated amino acid content, their corresponding improvements in AprE activity were less pronounced (Fig. 4c–d). Although glycine did not show significant changes in metabolomic profiles, it conferred a notable phenotype of increased enzyme activity. This may be attributable to its central position in the metabolic network and its involvement in multiple pathways—it functions not only as a fundamental amino acid for protein synthesis, but also as a key methyl donor in purine and thymidylate biosynthesis, and as a precursor for glutathione and heme production [32]. Therefore, even if its steady - state concentration does not decline markedly (possibly due to rapid compensatory endogenous synthesis or buffering by a large metabolic pool), restricted flux through this hub can still exert widespread effects on cellular physiology.

#### *3.3.3* Effect of reducing the central metabolism overflow of DM6E strain on AprE expression

Given the elevated intracellular concentrations of glycolic acid and acetate (Fig. 4e) in the AprE high-expressing strain DM6E/E*, we systematically evaluated multiple metabolic interventions to reduce the accumulation of organic carboxylic acids, including formate, acetate, oxalate and glyoxylate.

Regarding formate synthesis, genes *pflB* and *yrhE* were deleted from strain DM6E, and AprE activity of strains DM6E△pflB and DM6E△yrhE did not increase significantly (Fig. 4f). Concerning acetate synthesis, where pyruvate oxidase YdaP catalyzes the conversion of pyruvate to acetylphosphate [33], and its deletion strain DM6E△ydaP exhibited a 7.15% improvement in AprE activity over the control (Fig. 4g). For oxalate metabolism, deletion of oxalate decarboxylase gene *oxdC* generated strain DM6E△oxdC, which exhibited an AprE activity of 18,327 ± 681 U/mL and was 12.10% higher than DM6E (Fig. 4h). Regarding glyoxylate cycle regulation, we downregulated *aceA* transcription via λ_t0_ terminator insertion in *aceB-aceA* operon and enhanced *glcDF* expression by replacing its native promoter with the strong promoter PbacA (Fig. 4f–i). To identify strategies that mitigate intermediate overflow, recombinant strains were fermented in TB medium supplemented with 20 g/L glucose. Under these conditions, AprE activities of DM6*E*-AceBTA and DM6*E*-PbacA-glcDF reached 4444 ± 231 U/mL and 4168 ± 245 U/mL, which were 1.80 and 1.11 times higher than that of control strain DM6E, respectively (Fig. 4i).

### 3.4 Modular optimization of AprE expression strains

#### 3.4.1 Effect of energy supply pathway modification on the AprE-expressing strain DM6E1

**Fig. 5:**
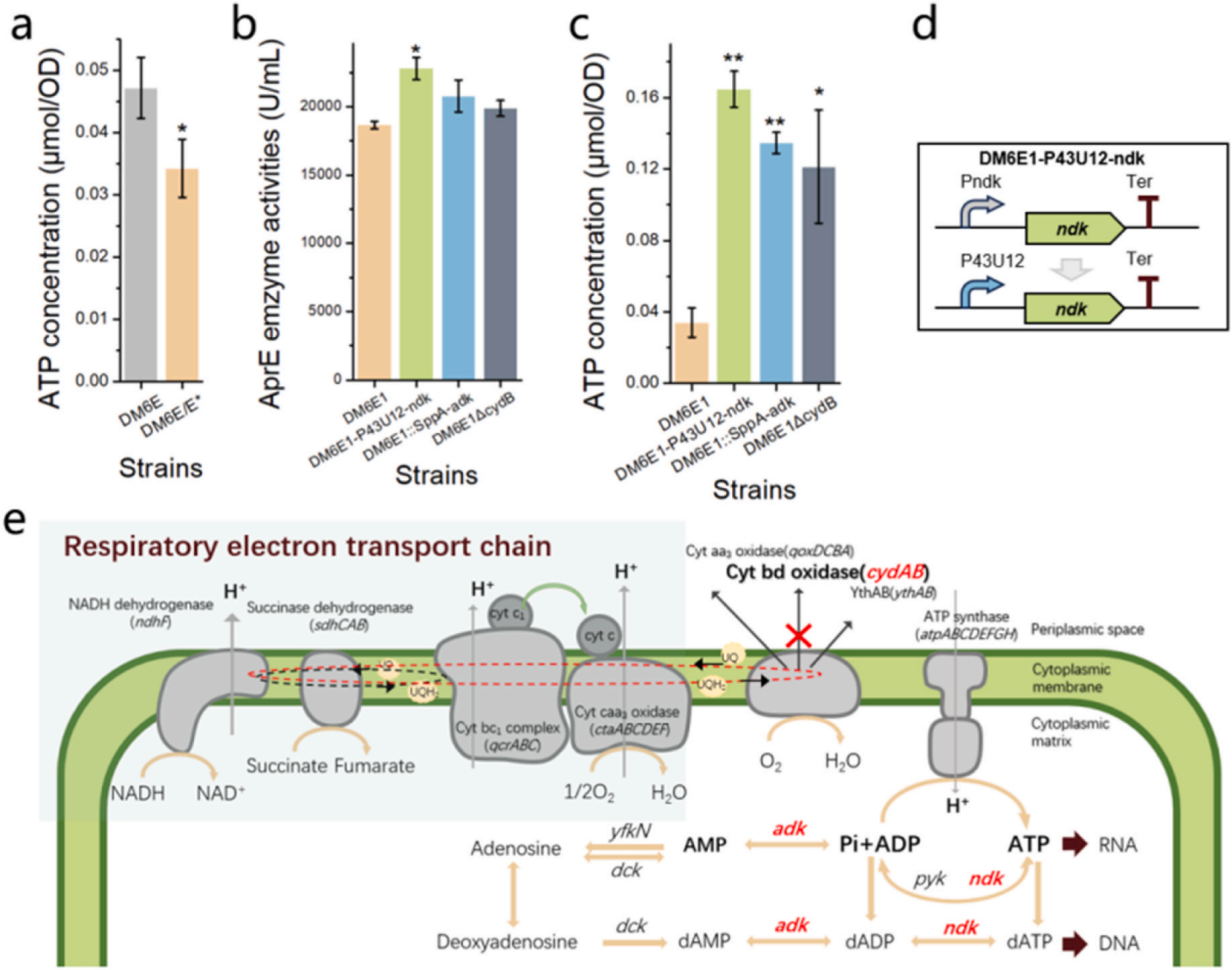
Effect of energy supply pathway modification of DM6E1 strain on AprE expression. **a:** ATP concentrations of DM6E and DM6E/E*. **b:** AprE activities. **c:** ATP concentrations. **d:** The modification of strains DM6E1-P43U12-ndk. **e:** Schematic illustration of energy supply module. Data are represented as the means of three replicates and error bars represent the standard deviations. *, *P* < 0.05; **, *P* < 0.01; ***, *P* < 0.001 indicate the significance levels between control and its mutant strains.

Energy supply constraints emerged in high-yield AprE strains, as evidenced by a 27.66% reduction in ATP concentration in DM6E/E* compared to DM6E (Fig. 5a). Given the relatively high pyrophosphate level in DM6E/E*, we speculate that energy supply is limited in these strains. Consequently, the energy metabolism module was modified and optimized.

Through replacing the native promoter of nucleoside diphosphate kinase gene *ndk* by promoter P43U12 in DM6E1, the activity of DM6E1-P43U12-ndk increased to 22,802 ± 792 U/mL, which is approximately 22.16% higher than that of DM6E1 (Fig. 5b), and ATP concentration per unit cell increased 3.83-fold, from 0.03 ± 0.01 to 0.17 ± 0.01 μmol (Fig. 5c). Although strengthening adenylate kinase gene (*adk*) expression and deleting cytochrome *bd* complex gene *cydB* also improved AprE expression, the effects were weaker than that of *ndk* overexpression (Fig. 5b). Hence, DM6E1-P43U12-ndk was chosen for further modification.

#### 3.4.2 Improving the performance of strain DM6E2 through cell membrane and cell wall engineering

Cell membrane and cell wall engineering, via directional modification of key components and surface proteins, can optimize strain functionality or confer new characteristics, such as material exchange, cell respiration and growth, while also influencing protein maturation and secretion [34]. This study implemented cell membrane and cell wall engineering of the AprE-producing strain from three perspectives, which are, enhancing membrane proteins involved in protein folding, optimizing cell wall synthesis and modification, and delaying cell autolysis.

**Fig. 6:**
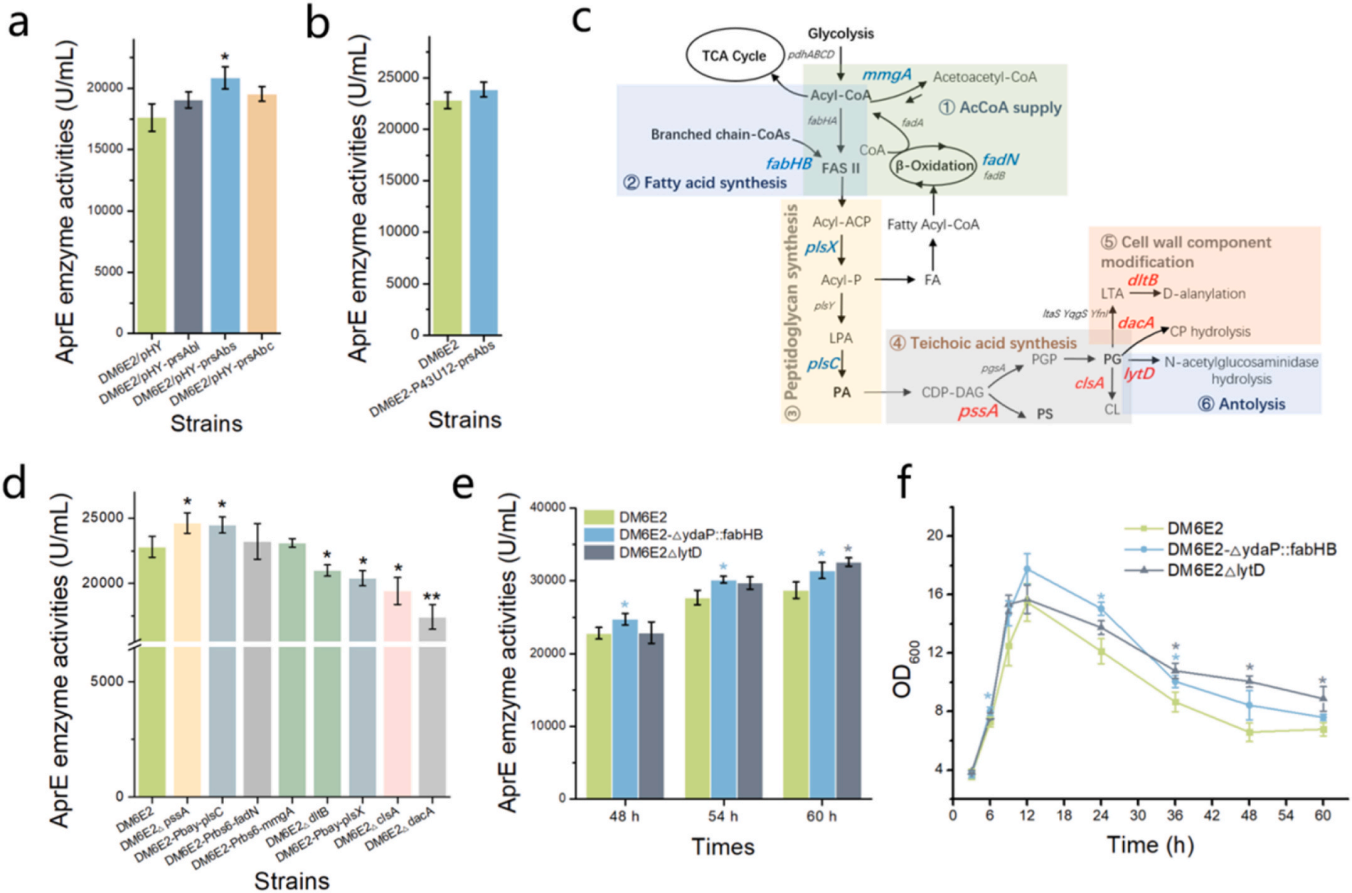
Effect of cell membrane and wall engineering on AprE expression. **a:** Effect of PrsA overexpression on AprE expression. **b:** Effect of PrsA^bs^ integration on AprE expression. **c:** Schematic of cell membrane and cell wall biosynthesis. Acyl-CoA, Acyl coenzyme A; FAS II, type II fatty acid synthase system; Acyl-ACP, Acyl-acyl carrier protein; Acyl-P, Acyl phosphate; LPA: Lysophosphatidic acid; PA, phosphatidic acid; CDP-DAG: Cytidine diphosphate diacylglycerol; PGP, Phosphatidylglycerol phosphate; PG, peptidoglycan; CL, Cardiolipin; CP, Carboxypeptidase; LTA, Lipoteichoic acid. **d:** AprE activities of cell membrane and cell wall engineered strains. **e:** AprE activities of DM6E2, DM6E2-△ydaPfabHB and DM6E2△lytD. **f:** Growth curve. Data are represented as the means of three replicates and error bars represent the standard deviations. *, *P* < 0.05; **, *P* < 0.01; ***, *P* < 0.001 indicate the significance levels between control and its mutant strains.

Firstly, we initially screened PrsA chaperones from *B. licheniformis*, *B. subtilis*, and *B. clausii*, designated PrsAbl, PrsAbs, and PrsAbc, respectively. These membrane-associated chaperones exert crucial functions in facilitating the folding and extracellular secretion of heterologous proteins. Among the strains overexpressing these PrsA variants, DM7E2/pHY-prsA^bs^ exhibited the highest AprE activity, followed by DM7E2/pHY-prsA^bc^ and DM7E2/pHY-prsA^bl^. The AprE activity of DM7E2/pHY-prsA^bs^ reached 20,851 ± 909 U/mL, which was 18.42% higher than that of the control strain DM7E2/pHY (17,608 ± 1199 U/mL) (Fig. 6a). Therefore, we selected *prsA*^*bs*^ for further engineering. It was expressed under the control of P43U12 promoter and integrated into the *nagB1* locus of DM6E2. The resulting engineered strain DM6E3 (DM6E2-nagB1P43U12-prsA^bs^) showed an even higher AprE activity of 23,867 ± 716 U/mL, which represented a 4.67% increase compared to DM6E2 (Fig. 6b).

Subsequently, we designed a series of modification strategies to optimize cell wall synthesis and modification. These strategies included enhancing the expression of acetyl-CoA supply genes *mmgA* and *fabN* [35], enhancing the expression of teichoic acid synthesis genes *plsX* and *plsC*, and deleting peptidoglycan synthesis bypass pathway genes *pssA*, *clsA*, *dacA* and *dltB* (Fig. 6c). Fermentation results showed that deletion of *pssA* increased AprE activity by 8.02%, while enhancement of *plsC* expression in DM6E2 increased AprE activity by 7.33% (Fig. 6d). The AprE activities of strains DM6E2△dltB, DM6E2-Pbay-plsX, DM6E2△clsA and DM6E2△dacA were all significantly lower than that of DM6E2, whereas the remaining engineered strains showed no significant changes in activity (Fig. 6d).

To improve biomass and delay autolysis of hosts, the fatty acid synthesis-related gene *fabHB* was integrated into the *ydaP* locus of DM6E2 strain, yielding the strain DM6E2△ydaP*fabHB*. In addition, the peptidoglycan degradation related gene *lytD* was deleted, generating the strain DM6E2△lytD (Fig. 6e). The AprE activities of these two strains reached 24,056 ± 947 U/mL and 22,882 ± 1474 U/mL, respectively, representing increases of 5.50% and 0.35% compared to the control strain DM6E2 (22,802 ± 792 U/mL) (Fig. 6e). Further biomass analysis showed that the overexpression of *fabHB* improved cell biomass (Fig. 6f), while deletion of *lytD* resulted in higher biomass and AprE activity when fermentation was extended to 54 h and 60 h (Fig. 6e–f).

### 3.5 Construction of a high-yield AprE-producing strain by modular engineering

**Fig. 7:**
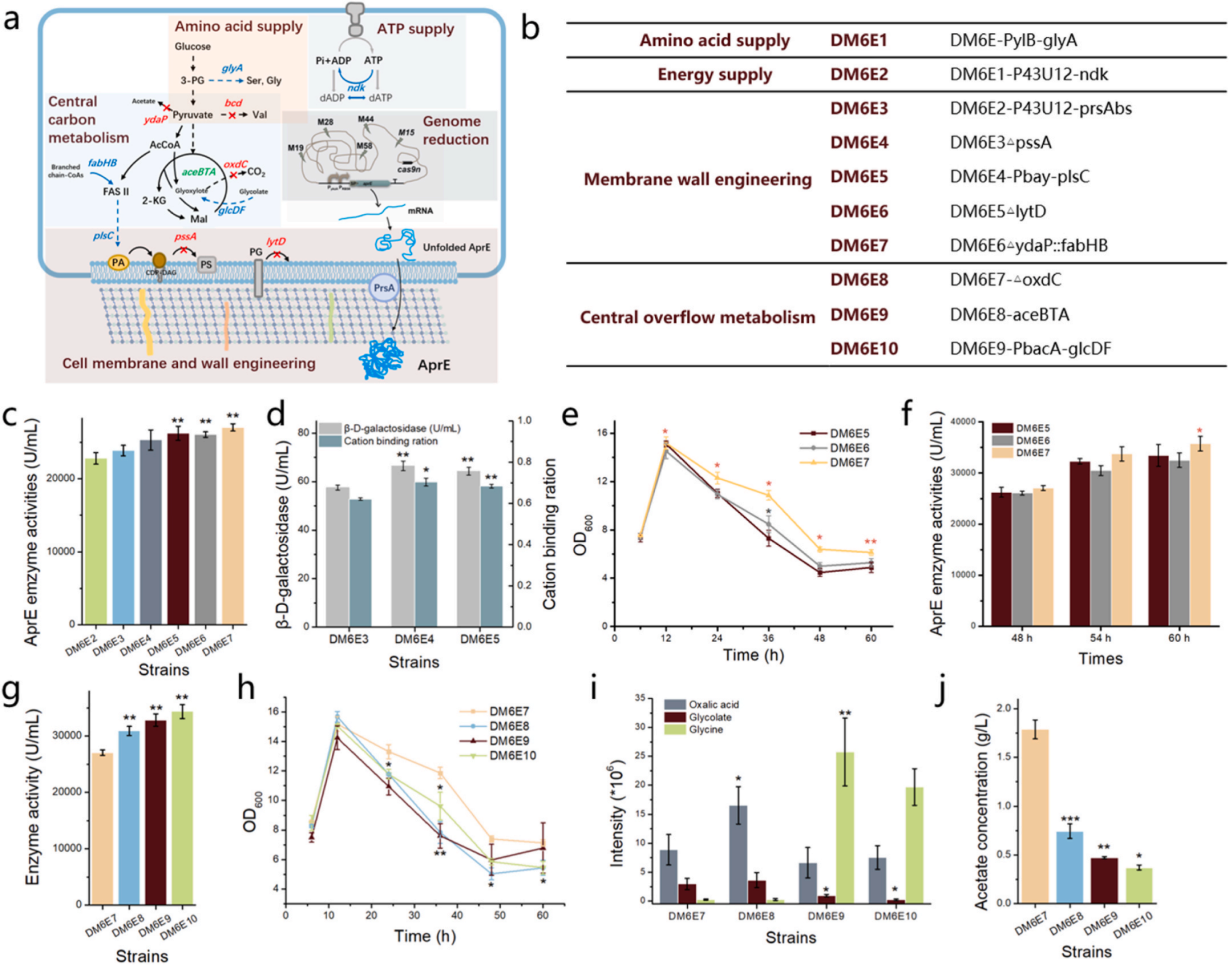
Modular engineering for construction of a high-yield AprE-producing strain. **a:** Construction diagram of DM6E10 chassis host. **b:** Information on engineered strains. **c:** AprE activities of superimposed strains in cell membrane and cell wall module. **d:** The cell surface net negative charge and membrane permeability of strains DM6E3, DM6E4 and DM6E5. **e:** Biomass of DM6E3, DM6E4 and DM6E5 in TB medium. **f:** AprE activities. **g:** AprE activities of overflow metabolism engineering strains. **h:** Biomass. **i:** The intensities of oxalic acid, glycolate and glycine. **j:** Acetate concentrations. Data are represented as the means of three replicates and error bars represent standard deviations. *, *P* < 0.05; **, *P* < 0.01; ***, *P* < 0.001 indicate the significance levels between control and its mutant strains.

Starting from the amino acid and energy supply-enhanced strain DM6E2 (DM6*E*-PylB-glyA-P43U12-ndk), we performed cell membrane and cell wall engineering. By sequentially integrating *prsA*^*bs*^ expression, deleting *pssA* and integrating *plsC* expression, we obtained strains DM6E3 (DM6E2-nagB1P43U12-prsA^bs^), DM6E4 (DM6E3△pssA) and DM6E5 (DM6E4-Pbay-plsC) (Fig. 7a–b). The AprE activities of these strains were measured as 23,867 ± 716 U/mL, 25,309 ± 1379 U/mL and 26,233 ± 655 U/mL, respectively (Fig. 7c). Our results in Fig. 7d showed that, compared with DM6E3, the cation binding ratio of DM6E4 and DM6E5 increased by 15.05% and 11.00% respectively; the cell membrane permeability increased by 13.38% and 10.16%, respectively. Subsequently, *lytD* was deleted and *fabHB* was integrated into DM6E5. The AprE activity of DM6E7 strain (DM6E5△lytD△ydaPfabHB) reached 27,043 ± 492 U/mL, which was 3.09% higher than that of DM6E5, approximately 18.60% higher than that of DM6E3 (Fig. 7c). Besides, the biomass of DM6E6 (DM6E5△lytD) and DM6E7 strains showed a continuous increase (Fig. 7e). When the fermentation time was extended to 60 h, the AprE activity of DM6E7 reached 35,735 ± 1451 U/mL, which was 6.18% higher than that of DM6E5 (Fig. 7f).

Then, the central metabolism overflow module was modified based on DM6E7, the *oxdC* gene was knocked out to obtain DM6E8 (DM6E7△oxdC). Compared with DM6E7, its AprE activity increased by 14.24%, reaching 30,896 ± 806 U/mL (Fig. 7g). Other physiological parameters showed that its biomass and by-product acetate concentration decreased, while glycine content increased (Fig. 7h–i). Since the weakening *aceA* had a better promoting effect on AprE activity in TB medium, DM6E9 (DM6E8-aceBTA) was constructed, and its AprE activity in AprE fermentation medium reached 32,831 ± 1084 U/mL (Figs. 4i and 7g). The *glcDF* gene was further enhanced to obtain strain DM6E10 (DM6E9-PbacA-glcDF), whose AprE activity reached 34,343 ± 1247 U/mL, approximately 27.00% higher than that of DM6E7 (Fig. 7g). Detection of organic carboxylic acids revealed that glycolate intensities in DM6E9 and DM6E10 were significantly reduced compared with those in DM6E8, while glycine intensities were increased (Fig. 7i). Furthermore, compared with DM6E7, the acetate content of DM6E8 showed a 58.39% reduction, and acetate contents of subsequently engineered strains DM6E9 and DM6E10 decreased progressively (Fig. 7j). This metabolic shift indicated enhanced glycolate consumption and carbon flux redirection toward the TCA cycle via acyl-CoA. Therefore, we obtained AprE high-yield host DM6E10 through modular engineering.

### 3.6 Fed-batch fermentation of AprE in a 5-L bioreactor

**Fig. 8:**
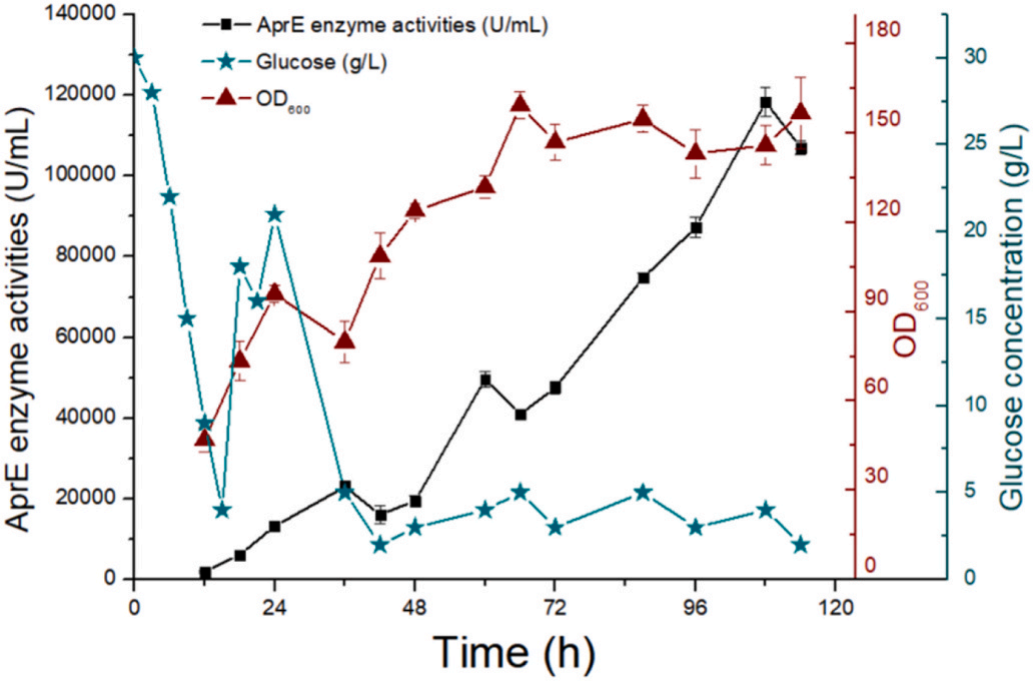
Time profiles of AprE activitiy of strain DM6E10 in a 5 L bioreactor. Data are represented as the means of three replicates and error bars represent standard deviations. *, *P* < 0.05; **, *P* < 0.01; ***, *P* < 0.001 indicate the significance levels between control and its mutant strains.

The strain DM6E10 was evaluated in a 5-L bioreactor for AprE fed-batch fermentation using a two-stage carbon source feeding strategy. The seed culture medium was inoculated at 10% (v/v) into the fermentation medium containing 30.0 g/L glucose. In the first stage, 150.0 mL of corn pulp (total mass 50.0 g) was added at 12 h and 24 h of fermentation, and the glucose concentration and biomass were monitored by sampling every 3 h. After the initial glucose was depleted, the second stage began, during which glucose was continuously fed to maintain a residual sugar concentration of approximately 15 g/L. When the OD_600_ exceeded 90, the carbon source was switched to 200.0 g of corn starch activated by α-amylase, and the entire fermentation process lasted 114 h. Process parameter monitoring showed that the biomass decreased at 36 h, followed by a secondary growth phase starting at 40 h, during which the biomass reached a peak of 159.4. The AprE activity slightly decreased at 40 h but ultimately achieved its maximum value of 107,100 ± 1671 U/mL at 108 h (Fig. 8).

## 4 Discussion

The global market size of industrial enzymes reached an estimated $7.42 billion in 2023, and is projected to continue its growth trajectory in the coming years, particularly within green manufacturing, biopharmaceutical and food industries. To meet the escalating demand, constructing efficient enzyme expression platforms via enzyme engineering and host modification has become a crucial strategy [36]. Current optimization approaches include codon optimization [37], increasing gene copy number [18], and enhancing expression elements [5] and secretion signal proteins [38]. As an excellent protein expression host, *B. licheniformis* is expected to be developed into a sustainable and robust cell factory. In this study, using AprE as a reporter protein, we systematically engineered efficient expression platforms for multiple secreted proteins in *B. licheniformis* DW2 by optimizing expression elements and cellular functional modules (Fig. 8). The relevant approaches not only improve protein expression levels but also provide valuable insights for advancing industrial enzyme production.

Subtle variations in codon preferences among phylogenetically close microorganisms are frequently overlooked [39]. In this study, we devised an evaluation scheme to identify restrictive codons. The application of this method to optimize the *aprE* gene resulted in a marked increase in its mRNA expression levels (Fig. 1b–c). Given that this method has not yet been validated on other proteins, subsequent work will assess its universality. In addition, other key factors such as the stability of RNA secondary structure, the accessibility of ribosome binding sites, and the translation-elongation rate can be comprehensively integrated to establish a more refined and universal codon optimization framework.

An ideal host chassis enables continuous cell growth, stable modular operation, and efficient overproduction of target products [40]. Streamlining non-essential metabolic pathways can reduce cellular metabolic burden and enhance product formation [41]. In this study, our reducted host chassis exhibited a remarkable 5.67-fold increase in AprE activity (Fig. 3c). Mechanism analysis revealed that the deletion of *ccdA1* increased intracellular ATP concentration, decreased cytochrome *c* content, and affected the production of disulfide bond proteins (Unpublished). These findings suggest that CcdA1 may suppress the expression of disulfide bond proteins, thereby reducing competition for secreted proteins and ultimately enhancing target protein expression (Unpublished).

This study employed metabolomics to systematically identify metabolic bottlenecks limiting AprE expression. Interestingly, amino acid supplementation experiments revealed that glycine addition was the most effective in enhancing AprE activity, rather than threonine, which exhibited the most pronounced changes in the metabolomic profile (Fig. 4a–b). We hypothesized that this outcome may be attributed to the broader physiological roles of glycine [32]. Subsequently, AprE activity was progressively enhanced through rational engineering transformation, including strengthening the supply of synthesis precursors, modifying the cell membrane and cell wall, and reducing central metabolic overflow. However, catabolite repression persisted during actual fermentation, indicating a need for further rational reconstruction of the host's transcriptional regulatory network [42]. Furthermore, future research will focus on the combinatorial module assembly and precise gene regulation to optimize synergistic interactions between modules. It is envisaged that the implementation of a genome-scale metabolic network (GSMN)-guided digital design platform will substantially accelerate the realization of these aims [43].

In conclusion, this study established universally applicable expression elements and reducted hosts, achieving 5.77-fold, 4.84-fold, and 1.31-fold enhancement in alkaline protease, nattokinase and chitinase activities, respectively. By combining metabolomics analysis with modular engineering, we systematically optimized precursor supply, modified the cell membrane and cell wall, and reduced central metabolism overflow in the AprE expression host. This comprehensive engineering approach yielded host DM6E10 with a final AprE activity of 34,343 ± 1247 U/mL, representing a 110% increase over DM6E (16,349 ± 316 U/mL). Scale-up in a 5 L bioreactor further elevated production to 107,100 ± 1671 U/mL, which is highly competitive in industrialization [44]. In summary, these strategies establish a high-performance *B. licheniformis* cell factory for recombinant protein expression.

## Data availability

Data will be made available on request.

## CRediT authorship contribution statement

**Qing Zhang:** Conceptualization, Formal analysis, Investigation, Methodology, Writing – original draft. **Mengyuan Zhang:** Methodology. **Zhihao Zhu:** Data curation. **Haijing Ma:** Software. **Fei Mo:** Methodology. **Jiaqi Lei:** Data curation. **Shisi He:** Investigation. **Yongqing Liao:** Software. **Shihao Yan:** Methodology. **Dongbo Cai:** Resources, Writing – review & editing. **Shouwen Chen:** Funding acquisition, Project administration, Supervision.

## Declaration of competing interest

The authors declare that they have no known competing financial interests or personal relationships that could have appeared to influence the work reported in this paper.
