# Supplementary material for: System reconstruction of Bacillus licheniformis for efficient expression of alkaline protease
Source: Synth Syst Biotechnol. 2026 Aug 1;17:26–38. doi: 10.1016/j.synbio.2026.07.005 (PMC13453031; doi:10.1016/j.synbio.2026.07.005)
Supplement: Supplementary file 1 [file mmc1.docx]

**System reconstruction of *Bacillus licheniformis* for efficient expression of alkaline protease**

Qing Zhang *^a^*, Mengyuan Zhang *^a^*, Zhihao Zhu *^a^*, Haijing Ma *^a^*, Fei Mo *^a^*, Jiaqi Lei *^a^*, Shisi He *^a^*, Yongqing Liao *^a^*, Shihao Yan *^a^*, Dongbo Cai *^a*^*, Shouwen Chen *^a,b*^*

*^a^ State Key Laboratory of Biocatalysis and Enzyme Engineering*, *Hubei Engineering Research Center for Microbial Cell Factories, College of Life Sciences, Hubei University, Wuhan, 430062, PR China*

*^b^	Hubei Jiangxia Laboratory, Wuhan 430200, China*

**Correspondence*: Prof. Shouwen Chen and Prof. Dongbo Cai

*Tel./fax.*: +86 027-88666081.

*E-mail address*: [mel212@126.com](mailto:mel212@126.com) (S. Chen) and [caidongbo@hubu.edu.cn](mailto:caidongbo@hubu.edu.cn) (D. Cai)

*Postal address*: 368 Youyi Avenue, Wuchang District, Wuhan 430062, Hubei, PR China

**Table S1 The strains used in this study.**

| **Strains** | **Characteristics** | **Sources** |
| --- | --- | --- |
| *E. coli DH5α* | F– Φ80d/lacZΔM15, Δ(lacZYA-argF) U169, recA1, endA1, hsdR17 (rK–, mK+), phoA, supE44, λ–, thi-1, gyrA96, relA1 | TaKaRa Co., Ltd |
| *B. licheniformis* DW2 | CCTCC M208065, wild type | CCTCC |
| DW2::cas9n (DM0) | DW2 with Cas9n interate strain | (Li et al., 2018) |
| DM1 | DW2::cas9n△M44 | This study |
| DM1/aprE | DM1/pHY-P43-aprE | This study |
| P97 | DM1/pHY-P43-aprE-P97^CCG^ | This study |
| S241 | DM1/pHY-P43-aprE-S241^TCG^ | This study |
| P116 | DM1/pHY-P43-aprE-P116^CCG^ | This study |
| S234 | DM1/pHY-P43-aprE-S234^TCG^ | This study |
| S26 | DM1/pHY-P43-aprE-S26^TCG^ | This study |
| P165 | DM1/pHY-P43-aprE-P165^CCG^ | This study |
| L355 | DM1/pHY-P43-aprE-L355^CUG^ | This study |
| P125 | DM1/pHY-P43-aprE-P125^CCG^ | This study |
| P344 | DM1/pHY-P43-aprE-P344^CCG^ | This study |
| L199 | DM1/pHY-P43-aprE-L199^CUG^ | This study |
| L14 | DM1/pHY-P43-aprE-L14^CUG^ | This study |
| P240 | DM1/pHY-P43-aprE-P240^CCG^ | This study |
| P87 | DM1/pHY-P43-aprE-P87^CCG^ | This study |
| P315 | DM1/pHY-P43-aprE-P315^CCG^ | This study |
| P150 | DM1/pHY-P43-aprE-P150^CCG^ | This study |
| S21 | DM1/pHY-P43-aprE-S21^TCG^ | This study |
| P162 | DM1/pHY-P43-aprE-P162^CCG^ | This study |
| P306 | DM1/pHY-P43-aprE-P306^CCG^ | This study |
| P97-P116 | DM1/pHY-P43-aprE-P97^CCG^-P116^CCG^ | This study |
| P97-S241 | DM1/pHY-P43-aprE-P97^CCG^-S241^TCG^ | This study |
| P97-S234 | DM1/pHY-P43-aprE-P97^CCG^-S234^TCG^ | This study |
| S241-P116 | DM1/pHY-P43-aprE-S241^TCG^-P116^CCG^ | This study |
| S241-S234 | DM1/pHY-P43-aprE-S241^TCG^-S234^TCG^ | This study |
| P116-S234 | DM1/pHY-P43-aprE-P116^CCG^-S234^TCG^ | This study |
| P97-S241-P116 | DM1/pHY-P43-aprE-P97^CCG^-S241^TCG^-P116^CCG^ | This study |
| P97-S241-S234 | DM1/pHY-P43-aprE-P97^CCG^-S241^TCG^-S234^TCG^ | This study |
| P97-S241-P116-S234 | DM1/pHY-P43-aprE-P97^CCG^-S241^TCG^-P116^CCG^-S234^TCG^ | This study |
| PykzAPrbs6 | DM1/pHY-PykzAPrbs6-aprE | This study |
| PR39 | DM1/pHY-PR55-aprE | This study |
| PR55 | DM1/pHY-PR39-aprE | This study |
| P4K | DM1/pHY-PykzAPrbs6-SP_AprE-P4K_-aprE | This study |
| T12L | DM1/pHY-PykzAPrbs6-SP_AprE-T12L_-aprE | This study |
| S11L-T12L-A13L | DM1/pHY-PykzAPrbs6-SP_AprE-S11L-T12L-A13L_-aprE | This study |
| S11L-T12L | DM1/pHY-PykzAPrbs6-SP_AprE-S11L-T12L_-aprE | This study |
| S26P | DM1/pHY-PykzAPrbs6-SP_AprE-S26P_-aprE | This study |
| S26Y | DM1/pHY-PykzAPrbs6-SP_AprE-S26Y_-aprE | This study |
| L5K | DM1/pHY-PykzAPrbs6-SP_AprE-L5K_-aprE | This study |
| P4K-A13L | DM1/pHY-PykzAPrbs6-SP_AprE-P4K-A13L_-aprE | This study |
| P4K-A13L-T12L | DM1/pHY-PykzAPrbs6-SP_AprE-P4K-A13L-T12L_-aprE | This study |
| P4K-T12L | DM1/pHY-PykzAPrbs6-SP_AprE-P4K-T12L_-aprE | This study |
| DM2 | DW2::cas9n△M44△M19 | This study |
| DM3 | DW2::cas9n△M44△M19△M28 |  |
| DM4 | DW2::cas9n△M44△M19△M28△M15 | This study |
| DM5 | DW2::cas9n△M44△M19△M28△M15△M58 | This study |
| DM6 | DW2::cas9n△M44△M19△M28△M15△M58△bcd | This study |
| DM6E | DW2::cas9n△M44△M19△M28△M15△M58△bcd::PykzAPrbs6-SP_AprE_-aprE | This study |
| DM0/E | DM0/pHY-PykzAPrbs6-SP_AprE-P4K-A13L_-aprE | This study |
| DM1/E | DM1/pHY-PykzAPrbs6-SP_AprE-P4K-A13L_-aprE | This study |
| DM1/E* | DM1/pHY-PykzAPrbs6-SP_AprE-P4K-A13L_-aprE-P97^CCG^-S241^TCG^-P116^CCG^ | This study |
| DM2/E* | DM2/pHY-PykzAPrbs6-SP_AprE-P4K-A13L_-aprE-P97^CCG^-S241^TCG^-P116^CCG^ | This study |
| DM3/E* | DM3/pHY-PykzAPrbs6-SP_AprE-P4K-A13L_-aprE-P97^CCG^-S241^TCG^-P116^CCG^ | This study |
| DM4/E* | DM4/pHY-PykzAPrbs6-SP_AprE-P4K-A13L_-aprE-P97^CCG^-S241^TCG^-P116^CCG^ | This study |
| DM5/E* | DM5/pHY-PykzAPrbs6-SP_AprE-P4K-A13L_-aprE-P97^CCG^-S241^TCG^-P116^CCG^ | This study |
| DM6/E* | DM6/pHY-PykzAPrbs6-SP_AprE-P4K-A13L_-aprE-P97^CCG^-S241^TCG^-P116^CCG^ | This study |
| DM6E/E* | DM6E/pHY-PykzAPrbs6-SP_AprE-P4K-A13L_-aprE-P97^CCG^-S241^TCG^-P116^CCG^ | This study |
| DM6E1 | DM6E-PylB-glyA | This study |
| DM6E2 | DM6E1-P43U12-ndk | This study |
| DM6E3 | DM6E2-P43U12-prsAbs | This study |
| DM6E4 | DM6E3△pssA | This study |
| DM6E5 | DM6E4-Pbay-plsC | This study |
| DM6E6 | DM6E5△lytD | This study |
| DM6E7 | DM6E6△ydaP::fabHB | This study |
| DM6E8 | DM6E7△oxdC | This study |
| DM6E9 | DM6E8-aceATB | This study |
| DM6E10 | DM6E9-PbacA-glcDF | This study |
| DM6E-Pbay-serA | *serA* gene is mediated by promoter Pbay in DM6E strain | This study |
| DM6E-Pbay-ysaA | *ysaA* gene is mediated by promoter Pbay in DM6E strain | This study |
| DM6E-P43U12-serC | *serC* gene is mediated by promoter P43U12 in DM6E strain | This study |
| DM6E-Pbay-floD | *folD* gene is mediated by promoter Pbay in DM6E strain | This study |
| DM6E△thiO | DM6E strain with *thiO* deletion | This study |
| DM6E2△pssA | DM6E2 strain with *pssA* deletion | This study |
| DM6E2-yadP::fabHB | DM6E2 strain integrating *fabHB* at the *ydaP* gene location | This study |
| DM6E2-Pbay-plsC | *plsC* gene is mediated by promoter Pbay in DM6E2 strain | This study |
| DM6E2-Prbs6-fadN | *fadN* gene is mediated by promoter Prbs6 in DM6E2 strain | This study |
| DM6E2-Prbs6-mmgA | *mmgA* gene is mediated by promoter Prbs6 in DM6E2 strain | This study |
| DM6E2△lytD | DM6E2 strain with *lytD* deletion | This study |
| DM6E2△dltB | DM6E2 strain with *dltB* deletion | This study |
| DM6E2-Pbay-plsX | *plsX* gene is mediated by promoter Pbay in DM6E2 strain | This study |
| DM6E2△clsA | DM6E2 strain with *clsA* deletion | This study |
| DM6E2△dacA | DM6E2 strain with *dacA* deletion | This study |
| DM6E-aceBTA | Terminater is added into *aceB-aceA* gene cluster in DM6E strain | This study |
| DM6E-PbacA-glcDF | *glcDF* gene is mediated by promoter PbacA in DM6E strain | This study |
| DM6E△pflBA | DM6E strain with *pflAB* deletion | This study |
| DM6E△yrhE | DM6E strain with *yrhE* deletion | This study |
| DW2dcas9/GFP | DW2dcas9/pHY-gRNA-amp::Pylb-GFP | This study |
| DM1/GFP | DM1/pHY-GFP | This study |

**Table S2 The plasmids used in this research.**

|  | Characteristics | Sources |
| --- | --- | --- |
| T_2_(2)-ori | *E. coli*–*Bacillus* shuttle vector; ori_Puc_/ori_ts_; temperature sensitive; Kan^r^ | Lab collection |
| T2-△bcd | T_2_(2)-ori derivative containing homologous arms for *bcd* knockout | (Liao et al. 2023) |
| T2-sipV::PP-aprE | T_2_(2)-ori containing homologous arms for *sipV* location and PykzAPrbs6-SPAprE-aprE expression box integration | This study |
| T2-PylB-glyA | T_2_(2)-ori containing homologous arms for PglyA deletion and PylB promoter insertion | This study  (Zhou et al.) |
| T2-P43U12-ndk | T_2_(2)-ori containing homologous arms for Pndk deletion and P43U12 promoter insertion | This study |
| T2-P43U12-prsAbs | T_2_(2)-ori containing homologous arms for *nagB1* location and P43U12-prsAbs expression box integration | This study |
| T2-△pssA | T_2_(2)-ori derivative containing homologous arms for *pssA* knockout | (Mo et al.2018) |
| T2-Pbay-plsC | T_2_(2)-ori containing homologous arms for PplsC deletion and Pbay promoter insertion | Lab collection |
| T2-△lytD | T_2_(2)-ori derivative containing homologous arms for *lytD* knockout | This study |
| T2-△ydaP::fabHB | T_2_(2)-ori containing homologous arms for *ydaP*location and P43U12-fabHB expression box integration | Lab collection |
| T2-△oxdC | T_2_(2)-ori derivative containing homologous arms for *oxdC* knockout | This study |
| T2-aceATB | T_2_(2)-ori containing homologous arms for *aceB-aceA* and lamda t_0_ terminater insertion | This study |
| T2-PbacA-glcDF | T_2_(2)-ori containing homologous arms for PglcDF deletion and P43U12 promoter insertion | (Wang et al. 2024) |
| T2-Pbay-serA | T_2_(2)-ori containing homologous arms for PserA deletion and Pbay promoter insertion | This study |
| T2-Pbay-ysaA | T_2_(2)-ori containing homologous arms for PysaA deletion and Pbay promoter insertion | This study |
| T2-P43U12-serC | T_2_(2)-ori containing homologous arms for PserC deletion and P43U12 promoter insertion | This study |
| T2-Pbay-folD | T_2_(2)-ori containing homologous arms for PfolD deletion and Pbay promoter insertion | (Zhou et al. 2023) |
| T2-△thiO | T_2_(2)-ori derivative containing homologous arms for *thiO* knockout | This study |
| T2-Prbs6-fadN | T_2_(2)-ori containing homologous arms for PfadN deletion and Prbs6 promoter insertion | This study |
| T2-Prbs6-mmgA | T_2_(2)-ori containing homologous arms for PmmgA deletion and Prbs6 promoter insertion | This study |
| T2-△lytD | T_2_(2)-ori derivative containing homologous arms for *lytD* knockout | This study |
| T2-△dltB | T_2_(2)-ori derivative containing homologous arms for *dltB* knockout | This study |
| T2-Pbay-plsX | T_2_(2)-ori containing homologous arms for PplsX deletion and Pbay promoter insertion | This study |
| T2-△clsA | T_2_(2)-ori derivative containing homologous arms for *clsA* knockout | This study |
| T2-△dacA | T_2_(2)-ori derivative containing homologous arms for *dacA* knockout | This study |
| T2-△pflBA | T_2_(2)-ori derivative containing homologous arms for *pflAB* knockout | This study |
| T2-△yrhE | T_2_(2)-ori derivative containing homologous arms for *yrhE* knockout | This study |
| pHY300PLK | *E. coli*–*Bacillus* shuttle vector; Amp^r^(*E. coli*) Tc^r^(both *E. coli* and *B. licheniformis*) | Lab collection |
| pHY-P43-aprE | pHY300PLK harboring P43 promoter, gene *aprE* | Lab collection |
| pHY-P43-aprE-P97^CCG^ | pHY300PLK harboring P43 promoter, gene *aprE*, whose P97 codon is mutated into CCG | This study |
| pHY-P43-aprE-S241^TCG^ | pHY300PLK harboring P43 promoter, gene *aprE*, whose S241 codon is mutated into TCG | This study |
| pHY-P43-aprE-P116^CCG^ | pHY300PLK harboring P43 promoter, gene *aprE*, whose P116 codon is mutated into CCG | This study |
| pHY-P43-aprE-S234^TCG^ | pHY300PLK harboring P43 promoter, gene *aprE*, whose S234 codon is mutated into TCG | This study |
| pHY-P43-aprE-S26^TCG^ | pHY300PLK harboring P43 promoter, gene *aprE*, whose S26 codon is mutated into TCG | This study |
| pHY-P43-aprE-P165^CCG^ | pHY300PLK harboring P43 promoter, gene *aprE*, whose P165 codon is mutated into CCG | This study |
| pHY-P43-aprE-L355^CUG^ | pHY300PLK harboring P43 promoter, gene *aprE*, whose L355 codon is mutated into CUG | This study |
| pHY-P43-aprE-P125^CCG^ | pHY300PLK harboring P43 promoter, gene *aprE*, whose P125 codon is mutated into CCG | This study |
| pHY-P43-aprE-P344^CCG^ | pHY300PLK harboring P43 promoter, gene *aprE*, whose P344 codon is mutated into CCG | This study |
| pHY-P43-aprE-L199^CUG^ | pHY300PLK harboring P43 promoter, gene *aprE*, whose L199 codon is mutated into CUG | This study |
| pHY-P43-aprE-L14^CUG^ | pHY300PLK harboring P43 promoter, gene *aprE*, whose L14 codon is mutated into CUG | This study |
| pHY-P43-aprE-P240^CCG^ | pHY300PLK harboring P43 promoter, gene *aprE*, whose P240 codon is mutated into CCG | This study |
| pHY-P43-aprE-P87^CCG^ | pHY300PLK harboring P43 promoter, gene *aprE*, whose P87 codon is mutated into CCG | This study |
| pHY-P43-aprE-P315^CCG^ | pHY300PLK harboring P43 promoter, gene *aprE*, whose P315 codon is mutated into CCG | This study |
| pHY-P43-aprE-P150^CCG^ | pHY300PLK harboring P43 promoter, gene *aprE*, whose P150 codon is mutated into CCG | This study |
| pHY-P43-aprE-S21^TCG^ | pHY300PLK harboring P43 promoter, gene *aprE*, whose S21 codon is mutated into TCG | This study |
| pHY-P43-aprE-P162^CCG^ | pHY300PLK harboring P43 promoter, gene *aprE*, whose P162 codon is mutated into CCG | This study |
| pHY-P43-aprE-P306^CCG^ | pHY300PLK harboring P43 promoter, gene *aprE*, whose P306 codon is mutated into CCG | This study |
| pHY-P43-aprE-P97^CCG^-P116^CCG^ | pHY300PLK harboring P43 promoter, gene *aprE* with codon optimization mutate P97^CCG^-P116^CCG^ | This study |
| pHY-P43-aprE-P97^CCG^-S241^TCG^ | pHY300PLK harboring P43 promoter, gene *aprE* with codon optimization mutate P97^CCG^-S241^TCG^ | This study |
| pHY-P43-aprE-P97^CCG^-S234^TCG^ | pHY300PLK harboring P43 promoter, gene *aprE* with codon optimization mutate P97^CCG^-S234^TCG^ | This study |
| pHY-P43-aprE-S241^TCG^-P116^CCG^ | pHY300PLK harboring P43 promoter, gene *aprE* with codon optimization mutate S241^TCG^-P116^CCG^ | This study |
| pHY-P43-aprE-S241^TCG^-S234^TCG^ | pHY300PLK harboring P43 promoter, gene *aprE* with codon optimization mutate S241^TCG^-S234^TCG^ | This study |
| pHY-P43-aprE-P116^CCG^-S234^TCG^ | pHY300PLK harboring P43 promoter, gene *aprE* with codon optimization mutate P116^CCG^-S234^TCG^ | This study |
| pHY-P43-aprE-P97^CCG^-S241^TCG^-P116^CCG^ | pHY300PLK harboring P43 promoter, gene *aprE* with codon optimization mutate P97^CCG^-S241^TCG^-P116^CCG^ | This study |
| pHY-P43-aprE-P97^CCG^-S241^TCG^-S234^TCG^ | pHY300PLK harboring P43 promoter, gene *aprE* with codon optimization mutate P97^CCG^-S241^TCG^-S234^TCG^ | This study |
| pHY-P43-aprE-P97^CCG^-S241^TCG^-P116^CCG^-S234^TCG^ | pHY300PLK harboring P43 promoter, gene *aprE* with codon optimization mutate P97^CCG^-S241^TCG^-P116^CCG^-S234^TCG^ | This study |
| pHY-PykzAPrbs6-aprE | pHY300PLK harboring PykzAPrbs6 promoter, gene *aprE* | This study |
| pHY-PR55-aprE | pHY300PLK harboring PR55 promoter, gene *aprE* | This study |
| pHY-PR39-aprE | pHY300PLK harboring PR39 promoter, gene *aprE* | This study |
| pHY-PykzAPrbs6-SPAprE-P4K | pHY300PLK harboring PykzAPrbs6 promoter, signal peptide SPAprE-P4K, gene *aprE* | This study |
| pHY-PykzAPrbs6-SPAprE-T12L | pHY300PLK harboring PykzAPrbs6 promoter, signal peptide SPAprE-T12L, gene *aprE* | This study |
| pHY-PykzAPrbs6-SPAprE-S11L-T12L-A13L | pHY300PLK harboring PykzAPrbs6 promoter, signal peptide SPAprE- S11L-T12L-A13L, gene *aprE* | This study |
| pHY-PykzAPrbs6-SPAprE-S11L-T12L | pHY300PLK harboring PykzAPrbs6 promoter, signal peptide SPAprE- S11L-T12L, gene *aprE* | This study |
| pHY-PykzAPrbs6-SPAprE-S26P | pHY300PLK harboring PykzAPrbs6 promoter, signal peptide SPAprE-S26P, gene *aprE* | This study |
| pHY-PykzAPrbs6-SPAprE-S26Y | pHY300PLK harboring PykzAPrbs6 promoter, signal peptide SPAprE-S26Y, gene *aprE* | This study |
| pHY-PykzAPrbs6-SPAprE-L5K | pHY300PLK harboring PykzAPrbs6 promoter, signal peptide SPAprE-L5K, gene *aprE* | This study |
| pHY-PykzAPrbs6-SPAprE-P4K-A13L | pHY300PLK harboring PykzAPrbs6 promoter, signal peptide SPAprE-P4K-A13L, gene *aprE* | This study |
| pHY-PykzAPrbs6-SPAprE-P4K-A13L-T12L | pHY300PLK harboring PykzAPrbs6 promoter, signal peptide SPAprE-P4K-A13L-T12L, gene *aprE* | This study |
| pHY-PykzAPrbs6-SPAprE-P4K-T12L | pHY300PLK harboring PykzAPrbs6 promoter, signal peptide SPAprE-P4K-T12L, gene *aprE* | This study |
| pHY-gRNA-M44 | pHY300PLK containing homologous arms and gRNA for M44 knockout | This study |
| pHY-gRNA-M19 | pHY300PLK containing homologous arms and gRNA for M19 knockout | This study |
| pHY-gRNA-M28 | pHY300PLK containing homologous arms and gRNA for M28 knockout | This study |
| pHY-gRNA-M15 | pHY300PLK containing homologous arms and gRNA for M15 knockout | This study |
| pHY-gRNA-M58 | pHY300PLK containing homologous arms and gRNA for M58 knockout | This study |
| pHY-P43-SPaprN-NK (also called N) | pHY300PLK harboring P43 promoter, signal peptide SPaprN and gene *aprN* | Lab collection |
| pHY-PykzAPrbs6-SPAprE-NK (also called NK*) | pHY300PLK harboring PykzAPrbs6 promoter, signal peptide SPAprE and gene *aprN* | This study |
| pHY-P43-SPno-chiT (also called ChiT) | pHY300PLK harboring P43 promoter, no signal peptide and gene *chiT* | Lab collection |
| pHY-PykzAPrbs6-SPAprE-chiT (also called ChiT*) | pHY300PLK harboring PykzAPrbs6 promoter, signal peptide SPAprE and gene *chiT* | This study |

**Table S3. The primers used in this study.**

| **Primers name** | **DNA sequence (5’-3’)** |
| --- | --- |
| T2-L | ATGTGATAACTCGGCGTA |
| T2-R | TCTGCTGAAGCCAGTTAC |
| T2-T5-F | TGGAGCTCGTAGAAAAGATCAA |
| T2-T5-R | GCTCTAGAACTAGTGGATCCCC |
| T2-sipV-F | TAACAGGCACGCCAAAAGCGG |
| SipV-PP-F | CTTTTGGCGTGCCTGTTAGAAATATTGATGTGACAC |
| SipV-aprE-R | CGCTAAGACTTCCAGGTGACGCAATAATGCCGTCGCACT |
| SipV-Tamyl-R | TCACCTGGAAGTCTTAGCGGTCA |
| ::sipV-KYF | GAGATTATTCGTAAAGCCGAGATG |
| ::sipV-KYR | CTGTCTCCCGTGTCTTTACCCG |
| T2-ndk-F1 | CTGCAGCCCGGGGGATCCTATGGAGCAAAACCTCAGAACCT |
| T2-ndk-R1 | GCATGAAATTGGAAATTCCGCAGAACTTTCGTTTGCCGATGTA |
| T2-ndk-F2 | TACATCGGCAAACGAAAGTTCTGCGGAATTTCCAATTTCATGC |
| T2-ndk-R2 | ACCATCACAAAGGTTTTTTCCATTATATATTCCTCCTTTCTAATAT |
| T2-ndk-F3 | ATATTAGAAAGGAGGAATATATAATGGAAAAAACCTTTGTGATGGT |
| T2-ndk-R3 | GATCTTTTCTACGAGCTCGCAGCCATTCTGATCCATATAA |
| T2-ndk-YF | TCAAAGCGAAGTGGGATAACC |
| T2-ndk-YR | CAACAAAGAAACTGTAATTGTCCAT |
| T2-NagB1-F | ATGATGTGATTGTGATGGCAGA |
| T2-NagB1-R | GAATAATTTTCACGTCGTTCTCC |
| NagB1::prsA-F | ACGACGTGAAAATTATTCTGATAGGTGGTATGTTTTCG |
| NagB1::prsA-R | CCATCACAATCACATCATTTTCCGTGGTGGGTGTATC |
| NagB1-YF | TAAAGGTCAAGAGGGAGGGTTC |
| NagB1-YR | TTATGATGGACCCTTTGCGG |
| T2-plsC-F1 | ATCCACTAGTTCTAGAGCCGAAAGCGGGACCCTAAG |
| T2-plsC-R1 | ATCGCAGGATGGCACTTC |
| T2-plsC-F2 | GAAGTGCCATCCTGCGAT |
| T2-plsC-R2 | TTATACATACAAATCTCC |
| T2-plsC-F3 | GGAGATTTGTATGTATAA |
| T2-plsC-R3 | TCTTTTCTACGAGCTCCAATTCAAAGCCTCCGTCAT |
| plsC-YF | GGACACATTCAGAGCTG |
| plsC-YR | ATGAGAGGGATCAGACCG |
| lytD-AF | TCTTTTCTACGAGCTCCAGTCGGTCGCCTTTGAGTA |
| lytD-AR | GATGGAAAGCCGAGGTCAACAAACAGGACACGCTCT |
| lytD-BF | AGAGCGTGTCCTGTTTGTTGACCTCGGCTTTCCATC |
| lytD-BR | ATCCACTAGTTCTAGAGCTACCGCACGTTGACAGCT |
| lytD-YF | CGGATCGTCCAGACCAAA |
| lytD-YR | AACCTGTTAAGGCGGATC |
| fabHB-AF | ACGGCCAGTGCCAAGCTTTAATAGTTGCCGCCACGA |
| fabHB-AR | TGAAATTGGAAATTCCGCATCACGTCCTCCTTTGTT |
| fabHB-BF | AACAAAGGAGGACGTGATGCGGAATTTCCAATTTCA |
| fabHB-BR | GCGCTTTTGATAAAGTTTTCATTATATATTCCTCCTTTCT |
| fabHB-CF | AGAAAGGAGGAATATATAATGAAAACTTTATCAAAAGCGC |
| fabHB-CR | TCCGTCCTCTCTGCTCTTTCAGGAAGCTGGCGCGCC |
| fabHB-DF | GGCGCGCCAGCTTCCTGAAAGAGCAGAGAGGACGGA |
| fabHB-DR | ATCTCCCTTGGCATCATACATCCGTCACAGTCTCAG |
| fabHB-EF | TATGATGCCAAGGGAGATCGT |
| fabHB-ER | ACAGCTATGACATGATTACGAATTCAAAGTCTGCGCGCTTAAG |
| fabHB-F | ATCGTTCAGCGGACAGG |
| fabHB-R | AAGCATAGTCCGCCGTT |
| OxdC-AF | TCTTTTCTACGAGCTCCAATTTCCTTGATTTTCGCA |
| OxdC-AR | CCTGGCTGGTTATTTCTTCTCTGATCGGCTGTGGAA |
| OxdC-BF | TTCCACAGCCGATCAGAGAAGAAATAACCAGCCAGG |
| OxdC-BR | ATCCACTAGTTCTAGAGCACAAGAACGAAAACCTCC |
| OxdC-KYF | TGAAAAGCCGCTCATACC |
| OxdC-KYR | GCAGGTGGTCCAGGTAGA |
| aceA-F1 | GGGCAATAAGAATGGTCG |
| aceA-F2 | GCAATAAGAATGGTCGGG |
| aceA-F3 | AGCAGTTGTAGGCGAGCA |
| aceA-F4 | TCTTTTCTACGAGCTCCACGCATACGCCAAACCTCT |
| aceB-R | CCCGCTAAAGACAAACCT |
| aceB-R2 | ATCCACTAGTTCTAGAGCGACGATGACAGTTCCCAA |
| aceA-seq-YF | CGGGACGAAATCCTCTGT |
| Toop-aceB-F | CCTAAGCTTTAGATAAAATTATATATTTTCATAAGCCGGTATC |
| Toop-aceB-F2 | GTCTTTCGACTGGGCCTAAGCTTTAGATAAAATTATATATTTTCATAAGCCGGTATC |
| Topp-aceA-R | TAGGCCCAGTCGAAAGACTGGGCCTTTTATGCTGAAAGAAAAACAACTCG |
| U12-serA-AF | ATCCACTAGTTCTAGAGCACAAAATCACAGCGAGCA |
| U12-serA-AR | CGAAAACATACCACCTATCACGTGTGGAAAAAGTGAGT |
| P43U12-serA-F | ACTCACTTTTTCCACACGTGATAGGTGGTATGTTTTCG |
| P43U12-serA-R | CAATACTCGAAACATTATATATTCCTCCTTTCT |
| P43U12-serA-BF | AGAAAGGAGGAATATATAATGTTTCGAGTATTG |
| U12-serA-BR | TCTTTTCTACGAGCTCCACGTCTTTGCGATGGTTTC |
| U12-serA-KYF | GGCAGGCAGAAACAATAG |
| U12-serA-KYR | CGCCCAAGTGAGGAGTCG |
| Pbay-ysaA-AF | ATCCACTAGTTCTAGAGCCCAACATAAAGCCCAGGA |
| Pbay-ysaA-AR | CTCGCCGAAATCGCAGGTGATAGAATGAAACGGGT |
| Pbay-ysaA-F | ACCCGTTTCATTCTATCACCTGCGATTTCGGCGAG |
| Pbay-ysaA-R | CAAAAAATACGGCTTTCATACAAATCTCCCCCTTTG |
| Pbay-ysaA-BF | CAAAGGGGGAGATTTGTATGAAAGCCGTATTTTTTG |
| Pbay-ysaA-BR | TCTTTTCTACGAGCTCCAGGAGGCTGAGGCAGTGTT |
| Pbay-ysaA-KYF | CCAACAGTGAAGAAACCG |
| Pbay-ysaA-KYR | GGGTGTAAGGTCAGTTTC |
| U12-serC-AF | ATCCACTAGTTCTAGAGCTTTGACGAAAATGGGGTG |
| U12-serC-AR | CGAAAACATACCACCTATCAGGTGCTTGTTGATGGAAG |
| P43U12-serC-F | CTTCCATCAACAAGCACCTGATAGGTGGTATGTTTTCG |
| P43U12-serC-R | GTTCGTTGTACGTATCATTATATATTCCTCCTTTCT |
| P43U12-serC-BF | AGAAAGGAGGAATATATAATGATACGTACAACGAAC |
| U12-serC-BR | TCTTTTCTACGAGCTCCACTGACGGTCCGAGGTTTT |
| U12-serC-KYF | CGGCAAGAGTTCCTGAGT |
| U12-serC-KYR | CAGCACGAGAGACAGCAT |
| ThiO-YF | CTCCAGCTTCTACGAATACAG |
| ThiO-YR | TCTCGCAAGGACGACATC |
| ThiO-UP-F | ATCCACTAGTTCTAGAGCTCTGGGATATGGCCTACCAA |
| ThiO-UP-R | GCTGAATGTTCATAGCGCTTTCTCATATGAAT |
| ThiO-DN-F | GCGCTATGAACATTCAGCTCAACGGCCGGAA |
| ThiO-DN-R | TCTTTTCTACGAGCTCCATCACAGCCGATCACTTCGAC |
| T2-fadN-F1 | TCCACTAGTTCTAGAGCCATAAAGTCCAGCACCCCG |
| T2-fadN-R1 | AGGAAGGATCAATGGTCAAGC |
| T2-fadN-F2 | GCTTGACCATTGATCCTTCCT |
| T2-fadN-R2 | GGGGAGGATCTTTCTGATAGGTGGTA |
| T2-fadN-F3 | TACCACCTATCAGAAAGATCCTCCCC |
| T2-fadN-R3 | TCTTTTCTACGAGCTCCAGCAAGTTCCAGTTCAGCA |
| fadN-YF | CGTCACAAGCAAGCCGTA |
| fadN-YR | GGTCCGCCATAAAATGTGC |
| T2-mmgA-F1 | ATCCACTAGTTCTAGAGCTCCTGCTCTTCCCTGGCA |
| T2-mmgA-R1 | GAAGGATCAATGGCGAGATCGG |
| T2-mmgA-F2 | CCGATCTCGCCATTGATCCTTC |
| T2-mmgA-R2 | GAGGATGTCAGTGATAGGTGGTAT |
| T2-mmgA-F3 | ATACCACCTATCACTGACATCCTC |
| T2-mmgA-R3 | TCTTTTCTACGAGCTCCAAATGCTCGCTGCCCTTAT |
| mmgA-YF | CGACCGTTAATTCGCC |
| mmgA-YR | TGGTCTGAAAGCGGAGG |
| T2-plsX-F1 | ATCCACTAGTTCTAGAGCGTTTATTACGGACGAGGA |
| T2-plsX-R1 | CGAAATCGCAGGGACTTACGCGCCAC |
| T2-plsX-F2 | CAGCAATTTTCATACAAATCTCCCCC |
| T2-plsX-R2 | GTGGCGCGTAAGTCCCTGCGATTTCG |
| T2-plsX-F3 | GGGGGAGATTTGTATGAAAATTGCTG |
| T2-plsX-R3 | TCTTTTCTACGAGCTCCATTTGACGCCCAGCACCTG |
| plsX-YF | ACCTAGTCATAATTGCGCG |
| plsX-YR | TCCGGCCATCAGAACC |
| T2-clsA-F1 | ATCCACTAGTTCTAGAGCGCTTCTCATCACAGTAAA |
| T2-clsA-R1 | CGACAGCAGATACGGGGCGG |
| T2-clsA-F2 | CCGCCCCGTATCTGCTGTCG |
| T2-clsA-R2 | TCTTTTCTACGAGCTCCACTGATGCCGAGGTCGTTA |
| clsA-YF | GCCACATGCTTGAGGTTA |
| clsA-YR | ATGCCGAGGTCGTTAGAT |
| T2-dltB-F1 | GAATACCAAATCAATCAG |
| T2-dltB-R1 | TTTCAGGGGATTGCTGTTCGCTT |
| T2-dltB-F2 | AAGCGAACAGCAATCCCCTGAAA |
| T2-dltB-R2 | CTTCGAAAATATCAATAT |
| dltB-YF | AAATCAAACTGCATGGCT |
| dltB-YR | TTCTGTAGCTGAAGTTTCG |
| dacA-YF | GCGGTATGGGTTCAGTGG |
| dacA-YR | CGGGATTGAAACAGCGT |
| pflA-YF1 | ATCCGCTCTCAAGGGCTC |
| pflB-YR | ACATCCGAGTCATCCCGT |
| pflA-AF | TCTTTTCTACGAGCTCCATCATAGAGGCGTACGAGT |
| pflA-AR | AGAGCGTGAAAACGGAGGGTTCAGAAGGTGGAGGTT |
| pflB-BF | AACCTCCACCTTCTGAACCCTCCGTTTTCACGCTCT |
| pflB-BR | ATCCACTAGTTCTAGAGCTTTCCGCTCGTCATCCTG |
| yrhE-YF | GCTCCGTTCCTCTGTTCA |
| yrhE-YR | TCATTATCGGCACCTCCA |
| yrhE-AF | TCTTTTCTACGAGCTCCAGCTTTTCGTTTTTCACTC |
| yrhE-AR | CACTTCCATACGCACTTTCGGAACATGGCATATTTG |
| yrhE-BF | CAAATATGCCATGTTCCGAAAGTGCGTATGGAAGTG |
| yrhE-BR | ATCCACTAGTTCTAGAGCAATAAAGGCAAGGAGGTG |
| aprE-S21-F | GTTGCTTTTTCGTCATCG |
| aprE-S21-R | CGATGACGAAAAAGCAAC |
| aprE-S26-F | CTGTCTCGGAGTTTGTAG |
| aprE-S26-R | CTACAAACTCCGAGACAG |
| aprE-S234-F | GCTAATTTGTCGTTAGGAAG |
| aprE-S234-R | CTTCCTAACGACAAATTAGC |
| aprE-S241-F | CTTCGCCATCGGCCACAC |
| aprE-S241-R | GTGTGGCCGATGGCGAAG |
| aprE-P97-F | GAACTCGATCCGGCGATTTC |
| aprE-P97-R | GAAATCGCCGGATCGAGTTC |
| aprE-P116-F | CAATCAGTGCCGTGGGGAAT |
| aprE-P116-R | ATTCCCCACGGCACTGATTG |
| aprE-P125-F | CAAGCCCCGGCTGCCCAT |
| aprE-P125-R | ATGGGCAGCCGGGGCTTG |
| aprE-P150-F | CACTCATCCGGACTTAAA |
| aprE-P150-R | TTTAAGTCCGGATGAGTG |
| aprE-P162-F | CTTTGTACCGGGGGAACC |
| aprE-P162-R | GGTTCCCCCGGTACAAAG |
| aprE-P165-F | GGAACCGTCCACTCAAGA |
| aprE-P165-R | TCTTGAGTGGACGGTTCC |
| aprE-P240-F | CTTCGCCGAGTGCCACAC |
| aprE-P240-R | GTGTGGCACTCGGCGAAG |
| aprE-P306-F | GTCGCACCGGGTGTAAAC |
| aprE-P306-R | GTTTACACCCGGTGCGAC |
| aprE-P315-F | CACATACCCGGGTTCAAC |
| aprE-P315-R | GTTGAACCCGGGTATGTG |
| aprE-P344-F | CAAAAGAACCCGTCTTGG |
| aprE-P344-R | CCAAGACGGGTTCTTTTG |
| tet-F | CTCCTTCTCCCATGGCTACTA |
| tet-R | TAGCCATGGGAGAAGGAGTCG |
| Tet-R | ATAGTAGCCATGGGAGAAGGAGTCGGTC |
| Tet-F | GACCGACTCCTTCTCCCATGGCTACTAT |
| seq-F | GGGGCTGTTTGCGTTTTT |
| seq-R | CCTCAGAGAGAATGGCGA |
| P4K-F | CAATGAAGAAAAAATTGGGGAAAATTG |
| P4K-R | CCAATTTTTTCTTCATTGATCCTTCCT |
| L5K-F | CAATGAAGAAACCGAAAGGGA |
| L5K-R | CTTTCGGTTTCTTCATTGATCCT |
| T12L-F | CAAGCCTGGCACTACTCA |
| T12L-R | TGAGTAGTGCCAGGCTTG |
| S11L-T12L-F | GTCGCACTTCTGGCACTACTCAT |
| S11L-T12L-R | TAGTGCCAGAAGTGCGACAATTTTC |
| S11L-T12L-A13L-F | GTCGCACTTCTGTTGCTACTCA |
| S11L-T12L-A13L-R | TAGCAACAGAAGTGCGACAATTT |
| S26P-F | GCACCGGCTGCTGAAGAA |
| S26P-R | TTCTTCAGCAGCCGGTGCGA |
| S26Y-F | GATCGCATATGCTGCTGAAG |
| S26Y-R | TCAGCAGCATATGCGATCGA |
| P4K-A13L-F | GCACCTTGCTACTCATTTC |
| P4K-A13L-R | AAATGAGTAGCAAGGTGC |
| M44-g-F | AGTGATAGCGGTACCATTGCCGTTAATCTCAGGCAATCGTTTTAGAGCTAGAAATAG |
| M44-g-R | TTGAATCTCGCCGAATAATGATGACGGTCCAGC |
| M44-AF | GCTGGACCGTCATCATTATTCGGCGAGATTCAA |
| M44-AR | GGGATGGGACTTGGAGATTGGTTTGCCTTCTCATT |
| M44-BF | AATGAGAAGGCAAACCAATCTCCAAGTCCCATCCC |
| M44-BR | TTTGCCCAAGCTTCTAGACGTCAGCACCGATGGA |
| M44-YF | TGCGACATTACCGTTCT |
| M44-YR | ATCGGAGCGGCTTTCG |
| M19-g1-F | AGTGATAGCGGTACCATTAAGGCGCGCCTTGTTGTTTGGTTTTAGAGCTAGAAATAG |
| M19-g1-R | GGCCACATACAGAGCGTCCGAAAAAAGCACCGACTCGG |
| M19-g2-F | CCGAGTCGGTGCTTTTTTCGGACGCTCTGTATGTGGCCGTTTTAGAGCTAGAAATAG |
| M19-g2-R | TGTTCGCAGTGACTATGATAATGATGACGGTCCAGC |
| M19-AF | GCTGGACCGTCATCATTATCATAGTCACTGCGAACA |
| M19-AR | AAGCAGCAGTACAATAGCGACTCTTGATTTTTTGCACCTG |
| M19-BF | CAGGTGCAAAAAATCAAGAGTCGCTATTGTACTGCTGCTT |
| M19-BR | TTTGCCCAAGCTTCTAGAATACGAGGTGAAATTCGG |
| M19-YF | AGAGCGACCAGGGCGATTC |
| M19-YR | CTGTAACCACAAATAACCCTT |
| M28-g1-F | AGTGATAGCGGTACCATTAAGCTTTCTTTTATCGGCAGGTTTTAGAGCTAGAAATAG |
| M28-g1-R | CGGTATTGGCGGCAAGCAGCAAAAAAGCACCGACTCGG |
| M28-g2-F | CCGAGTCGGTGCTTTTTTGCTGCTTGCCGCCAATACCGGTTTTAGAGCTAGAAATAG |
| M28-g2-R | GCCTGAACTACTGCCATTTTTAATGATGACGGTCCAGC |
| M28-AF | GCTGGACCGTCATCATTAAAAATGGCAGTAGTTCAGGC |
| M28-AR | GCGGGAAAAGGCTCTTCTTCGAAAAAACATCCCTTT |
| M28-BF | AAAGGGATGTTTTTTCGAAGAAGAGCCTTTTCCCGC |
| M28-BR | TTTGCCCAAGCTTCTAGATGATGCTCAGCGGGTTTG |
| M28-YF | AATACTGACAATTTCGCG |
| M28-YR | CAAGTCGTTGCCTGGATC |
| M58-g-F | AGTGATAGCGGTACCATTACGCTTCAGCCTGCCTTAGAGTTTTAGAGCTAGAAATAG |
| M58-g-R | ATCACCGCTTCCCAGTTCTCTAATGATGACGGTCCAGC |
| M58-AF | GCTGGACCGTCATCATTAGAGAACTGGGAAGCGGTGAT |
| M58-AR | TTGAGCGGTGCGGGTATCGTTAGGAAATCATTACGCATCA |
| M58-BF | TGATGCGTAATGATTTCCTAACGATACCCGCACCGCTCAA |
| M58-BR | TTTGCCCAAGCTTCTAGACTCCCTTTGCCGATTGAAA |
| M58-YF | CGGCAGAGCGTTTACATC |
| M58-YR | AAATGACGGAAAACGGAT |
| Amp-T5-F | CTGTCAGACCAAGTTTACTCATA |
| Amp-T5-R | GGGTTATTGTCTCATGAGCG |
| Pylb-GFP-PF | CGCTCATGAGACAATAACCCCATCGTCGAACGCGCTCC |
| Pylb-GFP-PR | CCCTTGCTCACCATACAAATCTCCCC |
| Pylb-GFP-GF | GGGGAGATTTGTATGGTGAGCAAGGG |
| Pylb-GFP-GR | GTAAACTTGGTCTGACAGGCCTTACCTATGCGAT |
| PP-NK-F | TCATCGATCGCATCGGCTGCCGGAAAAAGCAGTACA |
| PP-NK-R | GTAAACTTGGTCTGACAGTTATTGTGCAGCTGCTTG |
| pHY-PP-F | CTGTCAGACCAAGTTTACTCATA |
| pHY-PP-R | AGCCGATGCGATCGATGAACTAAAAGC |
| PP-chiT-F | TCATCGATCGCATCGGCTGCAAACGAGCCGGCGAAC |
| PP-chiT-R | GTAAACTTGGTCTGACATTATTCCTCTCCCGCAGT |

**Table S4. Codon composition of *aprE* gene (from *B. clausii* strain TCCC11004).**

| **Codons** | **Query** | **Codons** | **Query** | **Codons** | **Query** | **Codons** | **Query** |
| --- | --- | --- | --- | --- | --- | --- | --- |
| **GCA (A)** | 19 | **GCC (A)** | 10 | **GCG (A)** | 10 | **GCT (A)** | 14 |
| **TGC (C)** | 0 | **TGT (C)** | 0 | **GAC (D)** | 5 | **GAT (D)** | 5 |
| **GAA (E)** | 19 | **GAG (E)** | 9 | **TTC (F)** | 0 | **TTT (F)** | 6 |
| **GGA (G)** | 8 | **GGC (G)** | 9 | **GGG (G)** | 9 | **GGT (G)** | 11 |
| **CAC (H)** | 1 | **CAT (H)** | 7 | **ATA (I)** | 0 | **ATC (I)** | 3 |
| **ATT (I)** | 15 | **AAA (K)** | 7 | **AAG (K)** | 3 | **TTA (L)** | 9 |
| **TTG (L)** | 6 | **CTA (L)** | 3 | **CTC (L)** | 4 | **CTG (L)** | 0 |
| **CTT (L)** | 8 | **ATG (M)** | 5 | **AAC (N)** | 11 | **AAT (N)** | 13 |
| **CCA (P)** | 11 | **CCC (P)** | 0 | **CCG (P)** | 3 | **CCT (P)** | 3 |
| **CAA (Q)** | 9 | **CAG (Q)** | 3 | **AGA (R)** | 1 | **AGG (R)** | 0 |
| **CGA (R)** | 0 | **CGC (R)** | 3 | **CGG (R)** | 0 | **CGT (R)** | 4 |
| **AGC (S)** | 16 | **AGT (S)** | 4 | **TCA (S)** | 7 | **TCC (S)** | 4 |
| **TCG (S)** | 7 | **TCT (S)** | 7 | **ACA (T)** | 8 | **ACC (T)** | 1 |
| **ACG (T)** | 7 | **ACT (T)** | 5 | **GTA (V)** | 10 | **GTC (V)** | 9 |
| **GTG (V)** | 5 | **GTT (V)** | 12 | **TGG (W)** | 3 | **TAC (Y)** | 2 |
| **TAT (Y)** | 7 | **TAA (.)** | 1 | **TGA (.)** | 0 | **TAG (.)** | 0 |

**Table S5 Usage of synonymous codons in *B. licheniformis.***

| *Bacillus licheniformis* ATCC 14580 [gbbct]: 8375 CDS's (2436382 codons) | | | |
| --- | --- | --- | --- |
| [triplet] [frequency: per thousand] ([number]) | | | |
| UUU 29.3( 71366) | UCU 8.8( 21528) | UAU 20.3( 49483) | UGU 2.6( 6218) |
| UUC 16.0( 39017) | UCC 9.3( 22636) | UAC 13.5( 32899) | UGC 5.1( 12379) |
| UUA 13.9( 33868) | UCA 12.1( 29550) | UAA 2.0( 4807) | UGA 0.9( 2228) |
| UUG 17.6( 42970) | UCG 10.2( 24911) | UAG 0.5( 1340) | UGG 10.3( 24999) |
| CUU 22.4( 54534) | CCU 9.2( 22479) | CAU 13.4( 32542) | CGU 5.1( 12305) |
| CUC 14.9( 36290) | CCC 4.5( 10891) | CAC 8.4( 20513) | CGC 11.2( 27215) |
| CUA 3.1( 7513) | CCA 4.1( 10104) | CAA 16.6( 40452) | CGA 3.2( 7788) |
| CUG 25.7( 62525) | CCG 19.5( 47392) | CAG 19.2( 46754) | CGG 8.5( 20633) |
| AUU 29.4( 71544) | ACU 5.8( 14104) | AAU 18.7( 45636) | AGU 3.8( 9171) |
| AUC 34.8( 84889) | ACC 9.8( 23854) | AAC 19.2( 46745) | AGC 15.9( 38715) |
| AUA 7.2( 17442) | ACA 17.1( 41555) | AAA 49.1(119615) | AGA 9.6( 23276) |
| AUG 26.6( 64828) | ACG 19.4( 47377) | AAG 21.7( 52785) | AGG 6.4( 15588) |
| GUU 17.4( 42337) | GCU 15.7( 38294) | GAU 29.1( 70928) | GGU 9.9( 24233) |
| GUC 24.4( 59459) | GCC 21.8( 53052) | GAC 22.2( 53984) | GGC 26.9( 65528) |
| GUA 9.7( 23682) | GCA 18.4( 44917) | GAA 51.8(126258) | GGA 22.7( 55358) |
| GUG 16.1( 39127) | GCG 24.2( 58854) | GAG 21.8( 53193) | GGG 12.3( 29925) |

**Table S6 Restriction codon analysis^[1]^ of *aprE* gene expression in *B. lichienformis*.**

| **Codons** | **Amino Acid** | **Num** | **Prop. of Syn. Codons** | **Codon Usage (‰)** | **Ideal Prop.** | **Prop. of Syn. Codons/Ideal Prop.** | **Rstr. Codon** |
| --- | --- | --- | --- | --- | --- | --- | --- |
| **CCA** | Pro P | 11 | 64.71 | 28.95 | 4.10 | 15.78 | 1 |
| **AGU** | Ser S | 4 | 8.89 | 10.53 | 3.80 | 2.34 | 1 |
| **CUA** | Leu L | 3 | 10.00 | 7.89 | 3.10 | 3.23 | 1 |
| **UAA** | Ter* | 1 | 100.00 | 2.63 | 2.00 | 50.00 | 1 |
| **UGU** | Cys C | 0 | - | 0.00 | 2.60 |  | 1 |
| **GCA** | Ala A | 19 | 35.85 | 50.00 | 18.40 | 1.95 | 0 |
| **GAA** | Glu E | 19 | 67.86 | 50.00 | 17.40 | 3.90 | 0 |
| **AGC** | Ser S | 16 | 35.56 | 42.11 | 15.90 | 2.24 | 0 |
| **AUU** | Ile I | 15 | 83.33 | 39.47 | 29.40 | 2.83 | 0 |
| **GCU** | Ala A | 14 | 26.42 | 36.84 | 15.70 | 1.68 | 0 |
| **AAU** | Asn N | 13 | 54.17 | 34.21 | 18.70 | 2.90 | 0 |
| **GUU** | Val V | 12 | 33.33 | 31.58 | 17.40 | 1.92 | 0 |
| **GGU** | Gly G | 11 | 29.73 | 28.95 | 9.90 | 3.00 | 0 |
| **AAC** | Asn N | 11 | 45.83 | 28.95 | 19.20 | 2.39 | 0 |
| **GCC** | Ala A | 10 | 18.87 | 26.32 | 21.80 | 0.87 | 0 |
| **GCG** | Ala A | 10 | 18.87 | 26.32 | 24.20 | 0.78 | 0 |
| **GUA** | Val V | 10 | 27.78 | 26.32 | 9.70 | 2.86 | 0 |
| **GGC** | Gly G | 9 | 24.32 | 23.68 | 26.90 | 0.90 | 0 |
| **GGG** | Gly G | 9 | 24.32 | 23.68 | 12.30 | 1.98 | 0 |
| **GUC** | Val V | 9 | 25.00 | 23.68 | 24.40 | 1.02 | 0 |
| **UUA** | Leu L | 9 | 30.00 | 23.68 | 13.90 | 2.16 | 0 |
| **GAG** | Glu E | 9 | 32.14 | 23.68 | 21.80 | 1.47 | 0 |
| **CAA** | Gln Q | 9 | 75.00 | 23.68 | 16.60 | 4.52 | 0 |
| **GGA** | Gly G | 8 | 21.62 | 21.05 | 22.70 | 0.95 | 0 |
| **CUU** | Leu L | 8 | 26.67 | 21.05 | 22.40 | 1.19 | 0 |
| **ACA** | Thr T | 8 | 38.10 | 21.05 | 17.10 | 2.23 | 0 |
| **UCU** | Ser S | 7 | 15.56 | 18.42 | 8.80 | 1.77 | 0 |
| **UCA** | Ser S | 7 | 15.56 | 18.42 | 12.10 | 1.29 | 0 |
| **UCG** | Ser S | 7 | 15.56 | 18.42 | 10.20 | 1.53 | 0 |
| **ACG** | Thr T | 7 | 33.33 | 18.42 | 19.40 | 1.72 | 0 |
| **AAA** | Lys K | 7 | 70.00 | 18.42 | 49.10 | 1.43 | 0 |
| **UAU** | Tyr Y | 7 | 77.78 | 18.42 | 20.30 | 3.83 | 0 |
| **CAU** | His H | 7 | 87.50 | 18.42 | 13.40 | 6.53 | 0 |
| **UUG** | Leu L | 6 | 20.00 | 15.79 | 17.60 | 1.14 | 0 |
| **UUU** | Phe F | 6 | 100.00 | 15.79 | 29.30 | 3.41 | 0 |
| **GUG** | Val V | 5 | 13.89 | 13.16 | 16.10 | 0.86 | 0 |
| **ACU** | Thr T | 5 | 23.81 | 13.16 | 5.80 | 4.11 | 0 |
| **GAU** | Asp D | 5 | 50.00 | 13.16 | 29.10 | 1.72 | 0 |
| **GAC** | Asp D | 5 | 50.00 | 13.16 | 22.20 | 2.25 | 0 |
| **AUG** | Met M | 5 | 100.00 | 13.16 | 26.60 | 3.76 | 0 |
| **UCC** | Ser S | 4 | 8.89 | 10.53 | 9.30 | 0.96 | 0 |
| **CUC** | Leu L | 4 | 13.33 | 10.53 | 14.90 | 0.89 | 0 |
| **CGU** | Arg R | 4 | 50.00 | 10.53 | 5.10 | 9.80 | 0 |
| **AUC** | Ile I | 3 | 16.67 | 7.89 | 34.80 | 0.48 | 0 |
| **CCU** | Pro P | 3 | 17.65 | 7.89 | 9.20 | 1.92 | 0 |
| **CCG** | Pro P | 3 | 17.65 | 7.89 | 19.50 | 0.90 | 0 |
| **CAG** | Gln Q | 3 | 25.00 | 7.89 | 19.20 | 1.30 | 0 |
| **AAG** | Lys K | 3 | 30.00 | 7.89 | 21.70 | 1.38 | 0 |
| **CGC** | Arg R | 3 | 37.50 | 7.89 | 11.20 | 3.35 | 0 |
| **UGG** | Trp W | 3 | 100.00 | 7.89 | 10.30 | 9.71 | 0 |
| **UAC** | Tyr Y | 2 | 22.22 | 5.26 | 13.50 | 1.65 | 0 |
| **ACC** | Thr T | 1 | 4.76 | 2.63 | 9.80 | 0.49 | 0 |
| **CAC** | His H | 1 | 12.50 | 2.63 | 8.40 | 1.49 | 0 |
| **AGA** | Arg R | 1 | 12.50 | 2.63 | 9.60 | 1.30 | 0 |
| **CUG** | Leu L | 0 | 0.00 | 0.00 | 25.70 | 0.00 | 0 |
| **AUA** | Ile I | 0 | 0.00 | 0.00 | 7.20 | 0.00 | 0 |
| **CCC** | Pro P | 0 | 0.00 | 0.00 | 4.50 | 0.00 | 0 |
| **CGA** | Arg R | 0 | 0.00 | 0.00 | 3.20 | 0.00 | 0 |
| **CGG** | Arg R | 0 | 0.00 | 0.00 | 8.50 | 0.00 | 0 |
| **AGG** | Arg R | 0 | 0.00 | 0.00 | 6.40 | 0.00 | 0 |
| **UUC** | Phe F | 0 | 0.00 | 0.00 | 16.00 | 0.00 | 0 |
| **UAG** | Ter* | 0 | 0.00 | 0.00 | 0.50 | 0.00 | 0 |
| **UGA** | Ter* | 0 | 0.00 | 0.00 | 0.90 | 0.00 | 0 |
| **UGC** | Cys C | 0 | - | 0.00 | 5.10 |  | 0 |

**Table S7 The five large fragments conducive to protein expression.**

| **Large fragments** | **Gene clusters** | **Location** |
| --- | --- | --- |
| M44 | Bacitracin synthase gene cluster BacABC | 2547063..2589952 |
| M19 | Prophage region gene island M19 | 1397703..1421900 |
| M28 | Nonsense gene island M28 | 350347..370441 |
| M15 | Lichenysin synthase gene cluster LchAABCD | 402792..429000 |
| M58 | γ-PGA synthase gene cluster CapBCAE | 3929669..3932668 |

**Table. S8 Intracellular metabolite concentration of DM6E/E* strains.**

| **Category** | **Var ID (Primary)** | **DM6E/E* ave** | **Proportion of all compounds** | **Proportion of modular compounds** | **Sum** |
| --- | --- | --- | --- | --- | --- |
| Aromatic amino acids and their derivatives | Phenylethanol | 430720±177071 | 0.0014 | 0.4930 | 1 |
|  | Benzoic acid | 181257±104436 | 0.0006 | 0.2075 |  |
|  | Arbutin | 134905±88151 | 0.0004 | 0.1544 |  |
|  | Tyrosine | 116960±69706 | 0.0004 | 0.1339 |  |
|  | 3,4-dihydroxybenzoic acid | 9886±2636 | 0.0000 | 0.0113 |  |
| Fatty acid and glycerolipid | Stearic acid | 126044743±65034836 | 0.3960 | 0.5691 | 1 |
|  | Palmitic acid | 84905248±95077624 | 0.2668 | 0.3834 |  |
|  | Palmitoleic acid | 6706766±2175487 | 0.0211 | 0.0303 |  |
|  | Oleic acid | 2068964±1319851 | 0.0065 | 0.0093 |  |
|  | Linoleic acid | 1250919±2963489 | 0.0039 | 0.0056 |  |
|  | Cholesterol | 398506±126338 | 0.0013 | 0.0018 |  |
|  | Myristic acid | 94370±30997 | 0.0003 | 0.0004 |  |
| Central carbon metabolic compound | Glycolic acid | 20041530±28570979 | 0.0630 | 0.3548 | 1 |
|  | Enolpyruvate | 17886289±19588977 | 0.0562 | 0.3166 |  |
|  | Ethanolamine | 8887682±7146035 | 0.0279 | 0.1573 |  |
|  | Oxalic acid | 2148175±2392086 | 0.0067 | 0.0380 |  |
|  | Succinic acid | 1606349±3033518 | 0.0050 | 0.0284 |  |
|  | Gluconic acid | 1183254±489565 | 0.0037 | 0.0209 |  |
|  | Glycine | 1165305±571787 | 0.0037 | 0.0206 | 1 |
|  | Sucrose | 820697±234805 | 0.0026 | 0.0145 |  |
|  | Lactose | 481016±121935 | 0.0015 | 0.0085 |  |
|  | Galactose-6-phosphate | 478842±67675 | 0.0015 | 0.0085 |  |
|  | Maleic acid | 366141±292409 | 0.0012 | 0.0065 |  |
|  | Urea | 291244±81593 | 0.0009 | 0.0052 |  |
|  | Galactonic acid | 288610±111497 | 0.0009 | 0.0051 |  |
|  | Sulfuric acid | 269387±156024 | 0.0008 | 0.0048 |  |
|  | Maltose | 156978±47534 | 0.0005 | 0.0028 |  |
|  | Serine | 147500±154972 | 0.0005 | 0.0026 |  |
|  | Glucose-1-phosphate | 142563±93042 | 0.0004 | 0.0025 |  |
|  | Citric acid | 80970±181437 | 0.0003 | 0.0014 |  |
|  | Glucose | 36326±10642 | 0.0001 | 0.0006 |  |
|  | Phosphoethanolamine | 11278±1561 | 0.0000 | 0.0002 |  |
| Amino acids derived from pyruvate | Glycolic acid | 20041530±28570979 | 0.0630 | 0.6587 | 1 |
|  | Ethanolamine | 8887682±7146035 | 0.0279 | 0.2921 |  |
|  | Glycine | 1165305±571787 | 0.0037 | 0.0383 |  |
|  | Threonine | 171267±123904 | 0.0005 | 0.0056 |  |
|  | Serine | 147500±154972 | 0.0005 | 0.0048 |  |
|  | Phosphoethanolamine | 11278±1561 | 0.0000 | 0.0004 |  |
| Amino acids derived from 2-KG | Alanine | 2381158±2100874 | 0.0075 | 0.4905 | 1 |
|  | Valine | 1197489±825093 | 0.0038 | 0.2467 |  |
|  | Leucine | 807987±644198 | 0.0025 | 0.1664 |  |
|  | Isoleucine | 467987±221017 | 0.0015 | 0.0964 |  |
|  | Oxoproline | 3776225±1717888 | 0.0119 | 0.9116 |  |
|  | Glutamic acid | 366231±234024 | 0.0012 | 0.0884 |  |
| Sum | - | - | 1 | - | - |

1. Num: The number of amino acids in an AprE protein that contain this codon.

   Prop. of Syn. Codons: The proportion of synonymous codons.

   Codon Usage (‰): The relative frequency of a codon among all codons coding for the same amino acid (expressed as a millage).

   Ideal Prop. (‰): The ideal proportion of the codon in *B. licheniformis* strains.

   Rstr. Codon: Restriction codon.
